# Behavioral markers of motor reserve: stability, lateralization, and consistency

**DOI:** 10.3389/fnagi.2026.1921302

**Published:** 2026-09-30

**Authors:** David Vagni, Emanuela Principe, Melissa Pirrotta, Laura Catalano, Chiara Milasi, Morena De Francesco, Andreina Giustiniani, Chiara Arena, Francesco Scarfone, Angelo Quartarone, Domenico Bosco, Giovanni Pioggia, Antonio Cerasa

**Affiliations:** 1 Institute for Biomedical Research and Innovation (IRIB), National Research Council of Italy (CNR), Rome, Italy; 2 Department of Neurology, Pugliese Ciaccio Hospital, Catanzaro, Italy; 3 Institute for Biomedical Research and Innovation (IRIB), National Research Council of Italy (CNR), Messina, Italy; 4 Institute of Bioimaging and Complex Biological Systems (IBSBC), National Research Council of Italy (CNR), Catanzaro, Italy; 5 IRCCS Centro Neurolesi Bonino Pulejo, Messina, Italy

**Keywords:** healthy subjects, kinematic measure, motor performance, motor reserve, residual motor index

## Abstract

**Background:**

Motor reserve has been phenomenologically defined as the capacity to maintain motor performance despite aging-related or disease-related changes, but its objective kinematic basis remains insufficiently characterized. We operationalized motor reserve using measures from a sensor-equipped glove and tested whether they capture inter-individual differences beyond chronological aging.

**Methods:**

We enrolled 100 healthy adults (50 older and 50 younger) with a deliberately bimodal age distribution contrasting a young baseline with an older sample of interest. The glove recorded finger-level contact events during four tapping tasks (self-paced, maximum-velocity, synchronization to a 2-Hz metronome, and continuation after metronome withdrawal), each performed under right-hand, left-hand, and bilateral conditions. Motor reserve was assessed with the Motor Reserve Index questionnaire. Candidate kinematic indices were constructed to capture endogenous timing stability, externally paced velocity, bimanual coordination, touch-duration lateralization, and cross-context consistency (ClinicalTrials.gov database n° NCT07634055).

**Results:**

The central finding concerns which kinematic dimensions survive adjustment for chronological age. Aging affected a broad set of glove-derived parameters, most strongly under external pacing. After formal assessment of age confounding, three of the five candidate axes—endogenous timing stability, touch-duration lateralization, and cross-context consistency—retained an association with the Motor Reserve Index questionnaire (MRIq) independent of age, whereas externally paced velocity tracked age rather than reserve. A residual index (rMR) combining the three retained axes was associated with MRIq within the older group and predicted it out-of-sample (leave-one-out *r* = +0.50; *r* = +0.42 under nested cross-validation). Supporting analyses showed that the association was absent in the younger group, that the group-by-index interaction was significant, and that rMR was related to MRIq but not to cognitive reserve (CRIq) or to general neuropsychological performance.

**Conclusion:**

These findings provide initial evidence for a candidate kinematic index of motor reserve in healthy aging. Motor reserve was not reducible to global motor speed or chronological age but expressed as a multidimensional profile of timing stability, lateralized motor organization, and consistency across task contexts.

## 1 Introduction

Individual resilience to brain pathology has traditionally been framed within the theories of cognitive and brain reserve (Satz, 1993; Stern, 2002), which explain why individuals with comparable levels of neurodegeneration or structural damage can differ markedly in clinical and cognitive impairment. This paradigm has more recently been extended to the motor domain, as motor reserve (MR). Motor reserve refers to the brain’s capacity to maintain relatively preserved motor function despite underlying structural or functional damage to the motor network (Giustiniani and Quartarone, 2024). This resilience is thought to be driven by the flexible recruitment of alternative neural pathways, compensatory functional reorganization, and the structural integrity of parallel motor loops (Mitoma et al., 2020).

From a clinical point of view, MR has gained traction in the study of neurodegenerative diseases such as Parkinson’s disease (PD). For instance, patients with high pre-existing motor reserve can tolerate a significantly higher loss of dopaminergic neurons before developing overt clinical symptoms, and they often show better responses to therapeutic interventions or rehabilitation (Chung et al., 2020). While brain reserve represents a passive, structural entity (e.g., brain volume and synaptic density), motor reserve is fundamentally an active, dynamic process of functional compensation (Giustiniani and Quartarone, 2024). This clinical evidence rests, however, on an indirect measurement: In such studies, motor reserve is not measured but inferred from proxies of lifetime exposure—years of education, occupational demand, physical activity—which index the presumed causes of reserve rather than reserve itself.

The limitation of this approach is not the use of exposure proxies, but that a single exposure variable is treated as if it were the construct, with no measurement of motor behavior against which to anchor it. The Motor Reserve Index questionnaire (MRIq; Pucci et al., 2024) is the most structured instrument currently available for quantifying that exposure: It aggregates 17 items across six domains of lifetime physical activity from age 18 onward, weighting each activity by intensity, frequency, and years of practice, and it has been applied in clinical populations (Siciliano et al., 2022). It nonetheless remains a retrospective, self-reported measure of exposure. Although the MRIq represents the most structured instrument currently available for quantifying lifetime motor activity exposure, it remains based on retrospective self-report. This limitation highlights a broader challenge in the field: the absence of objective behavioral markers capable of capturing the motor manifestations of reserve. The present study addresses this gap by examining whether fine-grained kinematic measures derived from a sensor-equipped glove converge with MRIq scores and can provide objective behavioral signatures of motor reserve in healthy aging.

This comparison must contend with chronological age. Aging alters much of what a finger-tapping protocol measures: Movement slows, timing becomes more variable both within and across trials (Hultsch and MacDonald, 2004), the independence of individual fingers declines (Häger-Ross and Schieber, 2000), and the functional asymmetry between the hemispheres tends to reduce (Cabeza, 2002). Motor reserve and aging therefore act on overlapping kinematic parameters, and any measure that merely tracks age will correlate with any exposure variable that itself accumulates over the lifespan—MRIq included. Separating the two is a precondition for the primary question.

To address these limitations and identify candidate objective behavioral markers of motor reserve, the present study links lifetime motor activity, assessed through the Motor Reserve Index obtained from the MRIq, to kinematic measures of motor performance derived from a sensor-equipped glove, which records thumb-to-finger contact events separately for each finger and hand at millisecond resolution, yielding timing, duration, and asymmetry parameters for every task condition. We hypothesized that MR is expressed not merely as a single, global motor-speed advantage but rather as a multidimensional kinematic profile that captures fundamental properties of motor stability, temporal precision, and adaptability across varying task demands.

The secondary aim was to formally disentangle the influence of MR from the confounding effects of chronological aging. To this end, we mapped age-related declines across multiple kinematic parameters, tested for age-driven confounding, and implemented a residual-based analytics strategy. This approach operationalizes MR as the residual variability in performance that cannot be accounted for by chronological age alone, that is, effectively capturing an individual’s capacity to outperform their age-predicted functional baseline. Furthermore, we tested whether these objective kinematic markers converge with self-reported MRIq while remaining dissociable from cognitive reserve and general cognitive performance, thereby testing whether MR is empirically separable from cognitive reserve within the measures available here. Because the protocol is short, quantitative, and instrumented, an index of this kind could in future be evaluated as a candidate tool for patient stratification, although the present healthy-cohort data cannot establish that use.

Our hypotheses were therefore the following. First, we take motor reserve to be inter-individual variation in motor performance that is not attributable to chronological age. Second, we expected that once age is accounted for, only a subset of kinematic dimensions would retain an association with lifetime motor activity. The exploratory analysis of the older subsample was completed and locked before the younger group was recruited (§2.2, §2.6.1) (Supplementary material 1); on that basis, we predicted that the surviving subset would comprise timing stability, lateralized motor organization, and cross-context consistency and would not include global movement speed. Third, we expected these associations to be directional, with the predicted sign of each candidate axis fixed before testing; to be present in the older group but absent in the younger reference group; and to be specific to MRIq rather than extending to cognitive reserve or to general cognitive performance.

## 2 Materials and methods

Figure 1 summarizes the analytical pipeline, from the raw glove parameters to the residual index and the analyses used to validate it; the sections below follow the same order.

**Figure 1:**
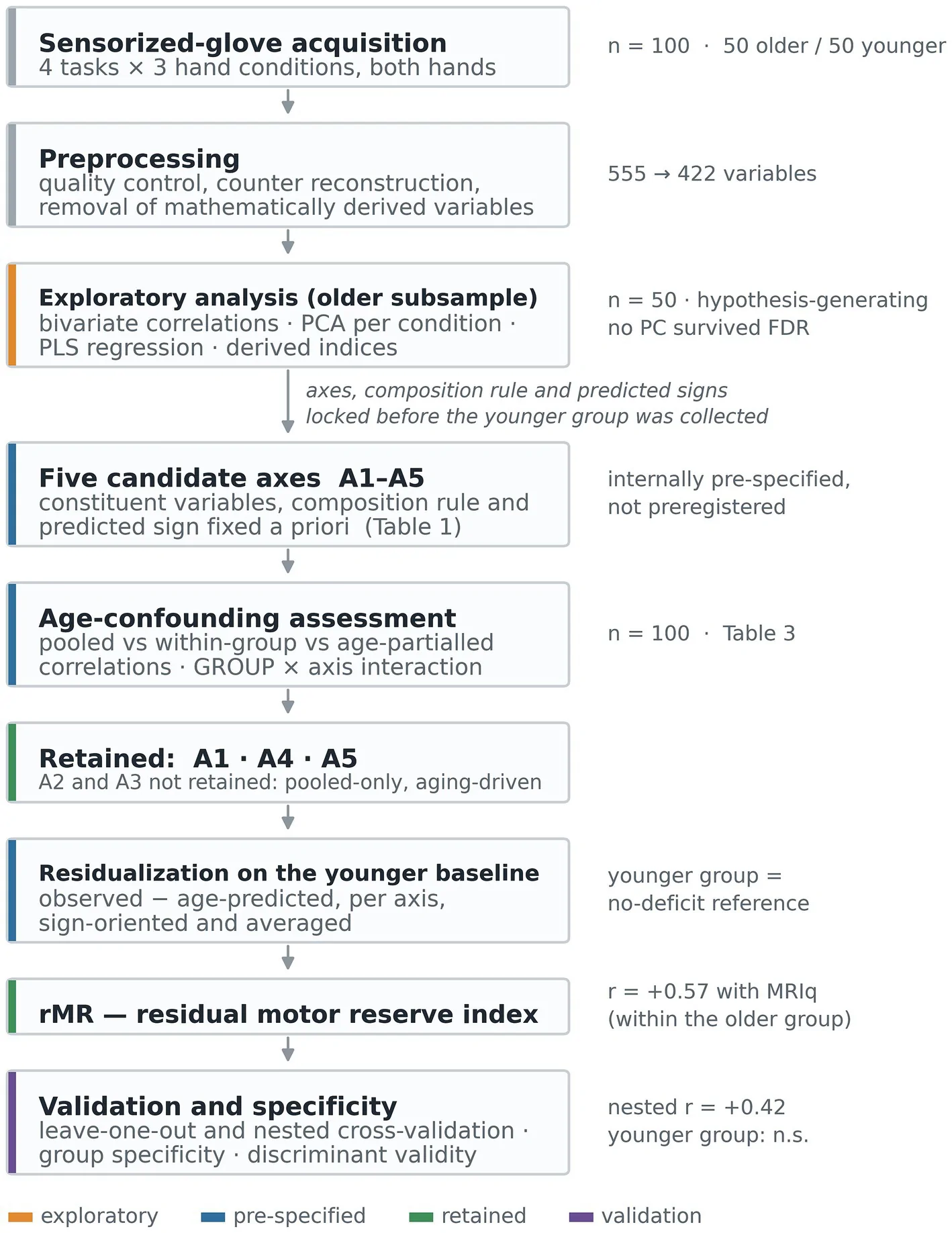
Analytical workflow. Raw glove parameters are reduced by preprocessing to the informative set, from which an exploratory analysis on the older subsample alone generated five candidate axes; the axes, their composition rule, and their predicted signs were locked before the younger group was collected. Each axis was then screened for age confounding, the three surviving axes were residualized against the younger-group age model, and the resulting index (rMR) was submitted to cross-validation, group-specificity, and discriminant analyses. The colored bar on the left of each box distinguishes exploratory from pre-specified steps; the annotations on the right give the sample and the corresponding table.

### 2.1 Participants

Between January and June 2026, we enrolled healthy controls in two temporally separated waves: The older sample was recruited first (January–February 2026), the five candidate axes and the analysis plan were then locked after preliminary analyses on the older subsample (March 2026), and the younger comparison group was recruited last (April–June 2026). We enrolled these participants at the Neurological Unit of the Azienda Ospedaliera Universitaria “Renato Dulbecco” in Catanzaro, through local advertisements distributed within the university and hospital settings. One hundred healthy adults (72 women and 28 men) took part in the study. The sample comprised 50 older adults (38 women and 12 men; age range 50–76 years, *M* = 60.6, SD = 7.0) and 50 younger adults (34 women and 16 men; age range 20–49 years at recruitment, with one participant turning 50 between recruitment and testing; *M* = 24.2, SD = 7.1). The age distribution was deliberately bimodal, with a sparse intermediate range (35–50 years), to sharpen the contrast between a young baseline and the older sample of interest. Mean education was 15.9 (SD = 2.0) years for older adults and 14.1 (SD = 1.9) years for younger adults; younger participants were largely current university students. The Edinburgh Handedness Inventory (Oldfield, 1971), administered with a 0–12 scoring scale, classified 94 participants as strict right-handers (score ≥ 7) and 6 as non-right-handers (score < 7); the latter were equally split between groups (3 per group).

Inclusion criteria were absence of self-reported neurological or psychiatric conditions, no current use of medications affecting motor or cognitive function, no upper limb conditions interfering with manual dexterity, and intact global cognitive function (corrected Mini-Mental State Examination ≥ 24). All participants completed the comprehensive neuropsychological battery described in §2.3. The corrected MMSE criterion was applied prospectively at screening and was met by all enrolled participants (older group: range 24.3–27.3; raw MMSE minimum 26), and in the older group the remainder of the battery was used to confirm the absence of cognitive impairment: all older participants performed within normal limits on the age- and education-corrected scores. No participant was excluded on neuropsychological grounds.

The study was approved by the Regional Ethics Committee of the Calabria Region (N° 114/2026) in accordance with the Declaration of Helsinki. The study has been registered in the ClinicalTrials.gov database (NCT07634055). All participants provided written informed consent prior to participation.

### 2.2 Motor kinematic protocol—sensor-equipped glove

Motor performance was assessed using a sensor-equipped glove that records the contact events between the thumb and the four fingers (Index → Middle → Ring → Little [IMRL sequence]) on each hand independently, providing finger-level timing parameters for each sequence of taps. The device is called HTS (Hand Test System) (Figure 2). This is a CE-marked medical device compliant with EU regulations developed by the company ETT S.p. A. (Genova, Italy).^1^

**Figure 2:**
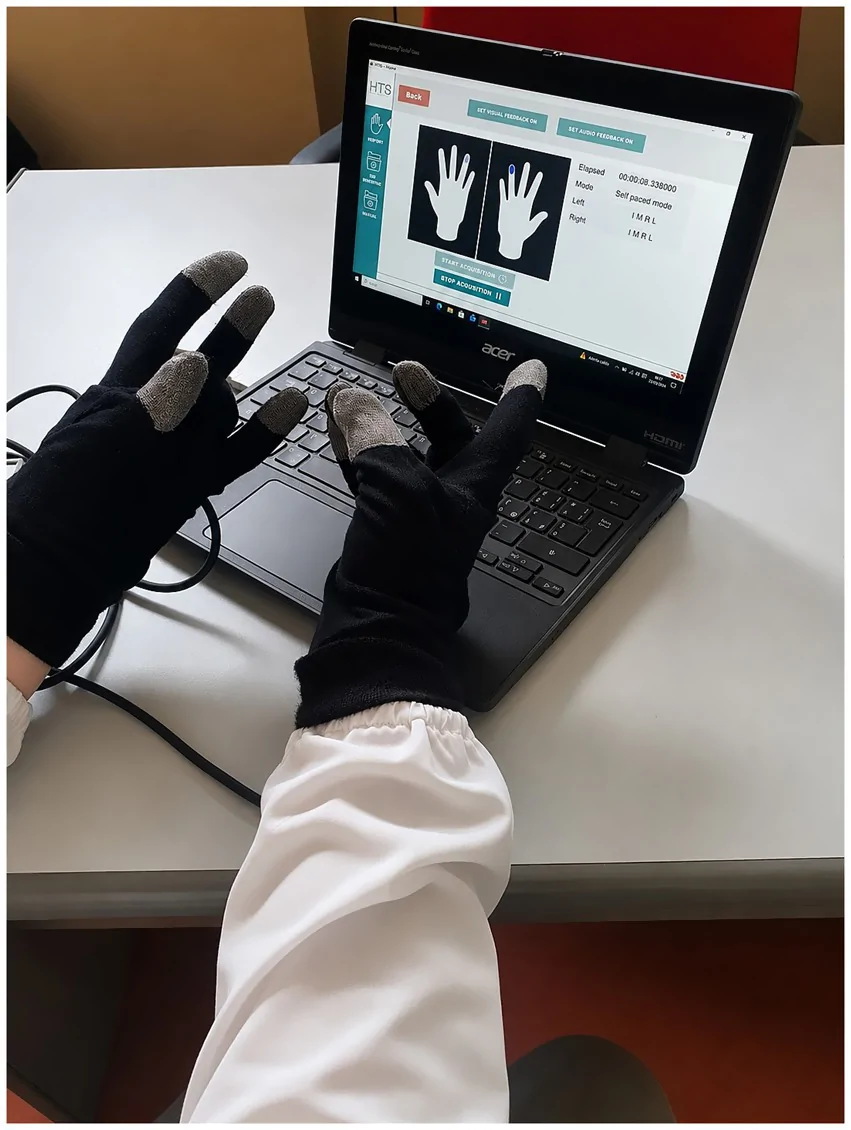
Sensor-equipped glove (HTS) device used for the evaluation of motor performance.

Each participant performed four motor tasks, each in three hand conditions (right-hand only, left-hand only, and bilateral), for a total of 12 acquisitions per subject. All acquisitions had a duration of 60 s. Participants wore the glove on both hands throughout the session. The tasks were performed in a fixed order:

- Self-Paced Movement (SPM): participants tapped repeatedly the four fingers in the IMRL sequence (index → middle → ring → little) at a self-chosen comfortable rhythm.
- Maximum velocity (MaxV): the same IMRL sequence was performed at the participant’s maximum achievable rate.
- Synchronization to a metronome (Metro): the same IMRL sequence was performed in synchrony with an auditory pacer at 2 Hz.
- Continuation (CONT): the same IMRL sequence was performed in synchrony with the 2 Hz metronome for the first 30 s; the metronome then ceased, and participants continued tapping at the learned rate for an additional 30 s without auditory feedback.

For each task and condition, the device extracted the following parameters per finger and per hand: touch duration (TD), inter-tapping interval (ITI), tapping rate, the ratio TD/ITI (a duty-cycle index of contact relative to interval), the count of correctly executed sequences and total taps, and (for tasks with the metronome) the time error of each tap relative to the closest pacing tone (TE), its absolute value (|TE|), and the percentage of advance versus (vs.) delay taps. For bilateral conditions in the metronome and continuation tasks, the device additionally computed inter-hand interval (IHI) onset and offset between corresponding finger taps of the two hands.

### 2.3 Neuropsychological assessment

All participants completed a neuropsychological battery covering global cognitive function, memory, attention, executive function, language, and praxis. The battery included the following: Mini-Mental State Examination (MMSE), Rey 15-words test (immediate recall, delayed recall, recognition), Corsi block-tapping test (forward and backward), Verbal span (forward and backward), Phonemic verbal fluency, Attention matrices, Trail Making Test parts A and B (and the B − A index of executive switching cost), Raven’s Colored Progressive Matrices (CPM), Frontal Assessment Battery (FAB), Ideomotor and Ideational apraxia tests, Free-hand drawing copy, Clock-drawing test, and the Hospital Anxiety and Depression Scale (HADS). Italian normative corrections for age and education were applied where available (Magni et al., 1996, for MMSE; Carlesimo et al., 1996, for Rey 15-words, phonemic verbal fluency, attention matrices and Raven’s Colored Progressive Matrices; Monaco et al., 2013, for forward and backward verbal and Corsi span; Giovagnoli et al., 1996, for Trail Making Test; Appollonio et al., 2005, for Frontal Assessment Battery). Tests showing ceiling effects in the present sample (MMSE, FAB, Clock test, Free-hand drawing copy, and Ideomotor and Ideational apraxia) were retained for description only and excluded from inferential analyses on cognitive performance. Manual lateralization was assessed with the Edinburgh Handedness Inventory (Oldfield, 1971; see §2.1); strict right-handers and non-right-handers were treated separately in sensitivity analyses (§2.6.8). The System Usability Scale (SUS; Brooke, 1996) was also administered after the motor protocol to assess perceived usability of the sensor-equipped glove. We applied the standard scoring procedure (raw 0–40 scale × 2.5) to obtain the conventional 0–100 score.

Neuropsychological evaluation included also the MRIq and CRIq scales. The Motor Reserve Index questionnaire (MRIq; Pucci et al., 2024) is a semi-structured questionnaire administered by a trained professional. It quantifies motor reserve through 17 items grouped in six areas covering lifetime physical activity from age 18 onward, including incidental and structured exercise, sport, manual occupational demands, and leisure-time motor activities. Each activity is scored as a function of intensity, frequency, and years of practice, and the items are aggregated into a total motor reserve score. Earlier use of the same instrument in spinocerebellar ataxia type 2 is reported in Siciliano et al. (2022). MRIq was administered to all 100 participants. The Cognitive Reserve Index questionnaire (CRIq; Nucci et al., 2012) was administered as an analogous measure of cognitive reserve, integrating items on education, working activity, and leisure activities. The instrument yields a total score with a normative population mean of 100 (SD = 15).

For inferential analyses involving cognitive performance, we constructed a neuropsychological composite as the mean of orientation-corrected z-scores for the 12 non-saturated cognitive measures: Rey 15-words (immediate, delayed, and recognition), Corsi (forward, backward), Verbal span (forward and backward), Phonemic fluency, Attention matrices, Trail Making A and B (sign-inverted), and Raven’s matrices. The HADS subscales were not included as they index mood rather than cognitive performance.

### 2.4 Data preprocessing

The raw dataset comprised 602 variables: 556 glove-derived motor parameters and 46 demographic, neuropsychological, and self-report variables. Data preprocessing included quality control, correction of acquisition inconsistencies, and removal of redundant variables (see Supplementary methods for the full pipeline). Of the 555 glove parameters, 133 were removed: 122 that are mathematically derived from others retained in the set (sums, means, and ratios—for example, hand-level rates replicated across individual fingers) and 11 standard errors that were almost perfectly collinear with the corresponding standard deviations (*r* ≈ 0.97). This redundancy is a property of the device export, which writes derived aggregates alongside the primitives they are computed from; retaining both would inflate the apparent dimensionality and distort the multiple-comparison correction. The 422 remaining variables are the informative set used throughout.

### 2.5 Construction of candidate motor reserve indices

To address the unfavorable ratio between motor variables (*n* = 422) and sample size (*n* = 100), we adopted a two-stage strategy (Figure 1). First, a preliminary exploratory analysis was conducted on the older-adult subsample (*n* = 50) using bivariate correlations, principal component analysis (PCA) per task condition, partial least squares regression, and derived indices (laterality, between-condition contrasts, and intra-individual variability (IIV) across conditions). This preliminary analysis is reported in Supplementary material 1 and identifies five candidate dimensions of motor performance that converged across analytical strategies in their association with MRIq. Second, based on these preliminary findings, we defined five composite indices using directly interpretable raw variables, with the predicted sign of the association with MRIq specified before testing them on the full dataset (*n* = 100). This pre-specification was internal to the project (not pre-registered on a public repository) and is documented in the analysis pipeline.

Three explicit criteria governed the passage from the exploratory output to the five composites. First, a dimension had to emerge concordantly from at least two of the exploratory strategies, so that no axis rests on a single analysis. Second, its constituent variables had to be directly interpretable raw parameters rather than component scores, so that each composite can be reproduced from the deposited dataset by applying the stated rule; this is why no principal component enters any axis, even though the components contributed to identifying the dimensions, and it is consistent with the fact that no component survived FDR correction against MRIq, either in the exploratory analysis (67 components, best pFDR = 0.17) (Supplementary material 1) or within the older group at the confirmatory stage (40 components, all pFDR ≥ 0.87). Third, the predicted sign had to be fixable in advance from the way the variables are constructed, so that each axis could be tested directionally. Table 1 documents, for each axis, its constituent variables, the composition rule, the rationale, the predicted sign, and the exploratory association that motivated it.

**Table 1:** Derivation of the five candidate axes.

Axis	Constituent variables	Composition rule	Rationale	Predicted sign	Exploratory evidence (older subsample, *n* = 50)
A1—Endogenous timing stability	SD of right-hand touch duration, self-paced tapping	z-score	Timing variability without an external pacer indexes endogenous timekeeping stability	−	*r* = −0.37, *p* = 0.008 = −0.37, *p* = 0.007
A2—Externally paced velocity	Tapping rate (right, left) and mean inter-tapping interval (right, left), metronome	mean z(rate) − mean z(interval)	Composite speed of externally paced tapping	+	Strongest constituent: left-hand rate *r* = +0.33, *p* = 0.020; composite *r* = +0.19
A3—Bimanual coordination	Inter-tapping-interval SD (right, left) and absolute inter-hand offset, bilateral metronome	mean of z-scores, offset in absolute value	Bilateral timing variability and looseness of inter-hand coupling	+ (direction observed in the exploratory analysis)	Constituents *r* = +0.13 to +0.18, all n.s.; composite *r* = +0.16
A4—Touch-duration lateralization	Laterality index (R − L)/(R + L) of touch duration: four-finger mean and middle finger, maximum-velocity and continuation	mean of the four z-scored laterality indices	Between-hand asymmetry as an index of preserved manual specialization	**–**	All four indices significant, *r* = −0.35 to −0.42, *p* ≤ 0.012; composite *r* = −0.49
A5—Cross-context consistency	Right-hand touch-duration SD and right- and left-hand mean inter-tapping interval, each in all four task conditions	within-individual SD across conditions of each z-scored metric, then mean	Intra-individual variability across task contexts (Hultsch and MacDonald, 2004)	**-**	Composite *r* = −0.40; strongest component *r* = −0.26

Constituent variables, composition rule, rationale, and predicted sign were fixed on the older subsample before the younger group was collected. The associations in the last column are computed in the same subsample from which the axes were derived and are therefore in-sample by construction: They document the signal that motivated each axis, not independent evidence for it. All constituent variables are z-scored before composition; r values are Pearson’s correlations with MRIq within the older group.

All composite indices were constructed by averaging the standardized values (z-scores) of the constituent variables. The predicted direction of the association with MRIq was specified *a priori* for each axis. A1, A4, and A5 are formulated so that higher values reflect greater variability, less hand specialization, or higher inter-hand offset, all of which indicate worse motor performance; their predicted association with MRIq is therefore negative. A2 and A3 were assigned a positive predicted sign: A2 because higher values reflect higher tapping velocity, and A3 because the bilateral-variability dimension from which it was derived correlated positively with MRIq in the exploratory analysis. The five indices are as follows:

- A1—Endogenous timing stability (predicted negative). The z-scored standard deviation of right-hand touch duration during self-paced tapping; a marker of timing variability in the absence of external pacing.
- A2—Externally paced velocity (predicted positive). A velocity index at the metronome: the standardized tapping rate (mean of both hands) minus the standardized inter-tap interval (mean of both hands); high values index fast tapping with short intervals.
- A3—Bimanual coordination (predicted positive, the direction observed in the exploratory analysis). The mean of the standardized bilateral inter-tap-interval variability (both hands) and the absolute inter-hand offset, at the bilateral metronome; high values reflect greater bilateral variability and looser inter-hand coupling.
- A4—Touch-duration lateralization (predicted negative). The mean standardized laterality index, LAT = (R − L)/(R + L), of touch duration, computed for the four-finger mean and the middle finger under maximum-velocity and continuation pacing (the middle finger being the most discriminating in the exploratory analysis).
- A5—Cross-context consistency (predicted negative). The mean standardized within-individual SD, across the four task conditions of three timing measures (right-hand touch-duration SD, and right- and left-hand mean inter-tap interval); high values indicate less stable performance across contexts (Hultsch and MacDonald, 2004).

Alongside the five candidate axes, we constructed a residual motor reserve index (rMR) following the Stern (2002, 2009) approach to cognitive reserve. The five axes and the analysis plan (predicted signs and the age-confounding reduction rule) were specified from the older-subsample exploration and locked before the younger group was collected (Supplementary material 1); the reduction to three followed this pre-planned rule, with the specific surviving axes (A1, A4, and A5) determined by the data. The index quantifies, for each older participant, the deviation of their motor performance from the value predicted by their age, using the younger group as the no-deficit baseline. For group-specificity analyses, the same signed residual was also computed for younger participants against the younger-baseline model and used only as a negative control, not interpreted as reserve in the young. For each of the three axes that passed the robustness checks (A1, A4, and A5; see §3.3), we estimated a linear regression of the index on age using only the younger subsample. The residual for each older participant was the observed value minus the value predicted by their age. The three z-standardized residuals were then averaged, with sign inversion so that positive rMR values mark performance better than expected for age, i.e., greater motor reserve.

The younger group is used as a functional baseline for the age trajectory of motor performance, not as a condition of maximal reserve. The baseline regression is estimated on age alone, and MRIq is never an input to the residualization. As expected, self-reported motor reserve is on average lower in younger than in older participants because MRIq is built up by lifetime motor exposure from age 18 onward; this between-group difference, however, cannot bias the residual, which captures only the deviation of an older individual’s performance from the value predicted by age.

Two important features of this operationalization follow. First, the linear baseline was fixed as part of the pre-specified plan, before the association between the resulting index and MRIq was examined; it was not chosen for the fit it produced. Second, the result does not depend on that choice. We re-estimated the baseline using a robust Theil–Sen regression, a Huber M-estimator, a quadratic age term, a model pooled across both groups, and an intercept-only model that removes the age term altogether. All five specifications yield within-older-group correlations between rMR and MRIq of *r* = +0.56 to +0.58, and all correlate at *r* ≥ 0.995 with the linear version (Supplementary Table S4). The intercept-only comparison is the informative one: Removing the age term entirely moves the correlation from *r* = +0.567 to *r* = +0.563. This follows from the age slope within the younger group not differing from zero, and it means that in the present design the residualization is close to a standardization of older performance against the younger mean. The Stern-style residual is conceptually motivated, and it is what a continuously sampled lifespan design would require, but in this bimodal design it is nearly inert. The younger-baseline assumption is therefore a reasonable first operationalization, not the only or the definitive way of separating aging from reserve.

### 2.6 Statistical analysis

The primary analysis is the association between the pre-specified candidate axes and MRIq, and the construction and evaluation of the composite index derived from the axes that survive it (§2.6.4, §2.6.6). The age-confounding analyses (§2.6.2, §2.6.3) establish which associations are attributable to chronological aging rather than to reserve and are a precondition for interpreting the primary analysis. The discriminant analyses (§2.6.7) test whether the resulting index is specific to motor rather than cognitive reserve. The robustness and sensitivity analyses (§2.6.5, §2.6.8) quantify how far the primary conclusions depend on analytical choices; they support the primary analysis but are not themselves findings, and their full detail is reported in Supplementary material.

All analyses were performed in Python 3.10.12 using the following libraries: pandas 2.3.3 and numpy 2.2.6 for data manipulation; scipy 1.15.3 for statistical tests; scikit-learn 1.7.2 for principal component analysis, regression, cross-validation, and model evaluation; statsmodels 0.14.6 for Huber robust regression, partial correlations, and multiple comparisons (Benjamini–Hochberg false discovery rate correction); pyreadstat 1.3.4 for reading the SPSS-format dataset; matplotlib 3.10.8 for figures. The full cleaned dataset is deposited at DOI: 10.17632/6j7yrhwxjb.1.

#### 2.6.1 Sample characterization

Continuous demographic and clinical variables were compared between groups using Welch’s *t*-test, and effect sizes are reported as Cohen’s d. Categorical variables (sex and handedness category) were compared using the chi-squared tests with Yates’ continuity correction for 2 × 2 tables. Distributional assumptions for inferential tests were verified via Shapiro–Wilk normality tests; non-normal variables were additionally tested non-parametrically (see Robustness).

#### 2.6.2 Aging effect map

To characterize the sensitivity of glove parameters to chronological aging, Cohen’s *d* for the difference between older and younger adults was computed for each of the 422 informative motor variables and summarized by task condition × parameter family, where a family groups parameters sharing a measurement construct (tapping rate, inter-tapping interval, touch duration, and time error). This map identifies which variables vary with aging and therefore which ones require the most careful separation between aging effects and reserve effects in subsequent analyses.

#### 2.6.3 Confounding by group: the Simpson check

Age and group are near-aliases in our bimodal design, so any pooled correlation between an age-sensitive motor variable and MRIq is likely inflated by the covariation of both quantities with age. We diagnosed Simpson-type confounding for each candidate axis by computing: (a) the Pearson correlation in the full sample (pooled *n* = 100), (b) within-group Pearson’s correlations (older; younger), (c) the partial correlation with MRIq controlling for age, and (d) the GROUP × axis interaction term in a regression model MRIq ~ axis + GROUP + axis: GROUP. The partial correlations were tested with the same one-tailed convention used for the directional axis tests (§2.6.4), evaluating each axis against its pre-specified sign; the GROUP × axis interaction term was tested two-tailed. A significant interaction (*p* < 0.05) flags genuine effect modification by group. Attenuated within-group correlations together with a near-zero partial-age correlation indicate a pooled-only effect driven by between-group covariation. Axes whose effect survives both partial correlation and testing within the older group are retained as robust to age confounding.

#### 2.6.4 Evaluation of the five candidate axes

Each of the five candidate axes was tested against MRIq using directional (one-tailed) Pearson’s correlations with the predicted sign, and Benjamini–Hochberg false discovery rate (FDR) correction was applied across the five hypotheses. Following the Simpson check (§2.6.3), the primary inferential analysis was performed within the older subsample (*n* = 50), where age is approximately constant and aging-related confounding is minimized. Pooled analyses on *n* = 100 are reported in §3.3, with explicit annotation of confounding.

#### 2.6.5 Bayes factors

Frequentist tests on small samples cannot distinguish evidence of absence from absence of evidence; we therefore computed Bayes factors (BF₁₀, the ratio of the evidence for the alternative hypothesis H1 over the null hypothesis H0) using the Bayesian information criterion (BIC) approximation: BF₁₀ = exp ((BIC₀ − BIC₁)/2), with BIC₀ for the intercept-only null model and BIC₁ for the linear model with the candidate index as predictor of MRIq within the older subsample (Wagenmakers, 2007). Conventional interpretation thresholds (Jeffreys, 1961) are as follows: 1 ≤ BF₁₀ < 3, anecdotal evidence for the alternative; 3 ≤ BF₁₀ < 10, moderate; 10 ≤ BF₁₀ < 30, strong; 30 ≤ BF₁₀ < 100, very strong; BF₁₀ ≥ 100, decisive. Values below 1 are read on the same scale after taking the reciprocal, so that BF₁₀ = 0.36 (BF₀₁ = 2.8) is anecdotal evidence for the null and BF₁₀ = 0.26 (BF₀₁ = 3.8) is moderate evidence for the null. Because the two lie on either side of the 1/3 boundary while differing little in magnitude, we do not treat them as qualitatively distinct (§3.4).

#### 2.6.6 Multivariate models

Three multivariate models were estimated within the older subsample, with MRIq as the dependent variable:

- the multivariate regression including all five candidate axes (5 predictors);
- the reduced regression including only the three robust axes (A1, A4, and A5);
- the simple regression on the rMR composite (1 predictor).

Model performance was assessed via in-sample R^2^ and via leave-one-out cross-validated R^2^LOO; all cross-validated values reported in the manuscript use the leave-one-out scheme. Significance of the cross-validated performance was assessed via permutation testing (1,000 permutations), shuffling MRIq labels and recomputing R^2^CV at each iteration. Bootstrap 95 and 99% confidence intervals for the bivariate correlation rMR × MRIq were computed via 2,000 resamplings of the older subsample with replacement.

#### 2.6.7 Discriminant validity

To check that the motor profile captured by the retained axes is distinct from cognitive reserve and from general cognitive performance, we contrasted the predictive performance of rMR (and of the multivariate three-axis model) for three parallel outcomes within the older subsample: MRIq, CRIq, and the neuropsychological composite. Differences between dependent correlations sharing the same predictor were tested with Steiger’s z in the form recommended for predictor-shared dependent correlations (Williams, 1959; Steiger, 1980).

#### 2.6.8 Sensitivity and robustness

The robustness of the primary result was assessed against several alternative specifications: (i) restricting the sample to strict right-handers; (ii) excluding multivariate outliers (subjects with > 40 motor variables at |z| > 3 within their group); (iii) including age, sex, and education as covariates in the regression on rMR; (iv) treating MRIq as an ordinal variable (tertiles) and testing group differences in rMR via Kruskal–Wallis and one-way ANOVA, with pairwise Mann–Whitney comparisons and a Spearman ordinal trend test; (v) replacing Pearson’s with Spearman, Kendall, and rank-based and log-transformed correlations; (vi) replacing ordinary least squares (OLS) regression with Huber robust regression (M-estimator); (vii) leave-one-subject-out inspection of the bivariate correlation. Threshold (segmented-regression) analysis was performed to test for non-linear relationships using an *F*-test against a single-line model.

## 3 Results

### 3.1 Sample characteristics

Demographic, neuropsychological, and self-report measures of the two age groups are summarized in Table 2. The two groups differed by design in age (Cohen’s *d* = 5.19). Raw MMSE scores were near ceiling in both groups and differed only slightly (*d* = −0.53); because global cognition was an inclusion criterion, this difference is not interpretable as an effect size (see the note to Table 2). Older participants also showed the typical age-related pattern of lower performance in memory (Rey 15-words: *d* = −0.83 immediate, *d* = −1.00 delayed), spatial and verbal short-term memory (Corsi forward *d* = −1.12, backward *d* = −1.16; verbal span backward *d* = −0.79), and fluid reasoning (Raven *d* = −1.24). The Trail Making Test was slower in older adults (TMT-A *d* = +0.83). Mean education was higher in the older group (15.9 vs. 14.1 years, *d* = 0.96) because younger participants were largely current university students. Sex distribution did not differ between groups (38F/12M older vs. 34F/16M younger, χ^2^ = 0.45, *p* = 0.50), nor did the proportion of strict right-handers (47/3 in each group, *p* > 0.99). The Frontal Assessment Battery and Hospital Anxiety and Depression scales were comparable across groups; no participant reached clinical cutoffs for frontal-executive dysfunction or mood symptoms.

**Table 2:** Sample characteristics by group.

Variable	Older (*n* = 50)	Younger (*n* = 50)	*p*	Cohen’s *d*
*N*	50	50	–	–
Age, years (mean ± SD; median [IQR])	60.6 ± 7.0; 60 [55–65]	24.2 ± 7.1; 21 [21–24]	<0.001	+5.19
Education, years	15.9 ± 2.0	14.1 ± 1.9	<0.001	+0.96
Sex, F/M	38/12	34/16	0.504	–
Handedness, R/mixed	47/3	47/3	>0.99	–
Edinburgh handedness score (0–12)	11.1 ± 2.7	10.6 ± 2.2	0.37	+0.18
MRIq	62.3 ± 13.0 [21.3–98.7]	48.9 ± 12.9 [27.8–73.3]	<0.001	+1.04
CRIq	119.8 ± 15.4 [98.0–166.0]	94.3 ± 6.4 [85.0–112.0]	<0.001	+2.15
MMSE (raw)	28.6 ± 0.7	28.9 ± 0.4	0.010	−0.53
Rey 15-words, immediate	46.4 ± 7.6	52.5 ± 7.3	<0.001	−0.83
Rey 15-words, delayed	9.6 ± 2.5	11.8 ± 1.8	<0.001	−1.00
Corsi span, forward	4.9 ± 0.8	5.9 ± 0.9	<0.001	−1.12
Corsi span, backward	4.5 ± 0.9	5.5 ± 0.8	<0.001	−1.16
Verbal span, forward	7.1 ± 1.1	7.2 ± 1.2	0.73	−0.07
Verbal span, backward	4.6 ± 1.0	5.4 ± 1.0	<0.001	−0.79
Phonemic verbal fluency	38.5 ± 7.0	41.2 ± 7.6	0.072	−0.36
Attentive matrices	52.8 ± 5.5	55.3 ± 3.7	0.011	−0.52
Trail making test-A (s)	41.4 ± 12.7	32.3 ± 8.9	<0.001	+0.83
Trail making test-B (s)	91.3 ± 35.4	78.4 ± 18.3	0.024	+0.46
Trail making test B − A (s)	49.9 ± 27.9	46.1 ± 16.3	0.406	+0.17
Raven’s CPM	31.0 ± 3.6	34.4 ± 1.4	<0.001	−1.24
Frontal assessment battery	17.5 ± 0.8	17.6 ± 0.6	0.256	−0.23
HADS anxiety	6.2 ± 3.6	6.7 ± 3.1	0.482	−0.14
HADS depression	3.0 ± 2.7	2.7 ± 2.6	0.541	+0.12
SUS (0–100)	77.4 ± 7.5	69.3 ± 8.3	<0.001	+1.03

IQR, interquartile range. MMSE values are raw scores. The inclusion criterion (age/education-corrected MMSE ≥ 24) and the near-ceiling distribution make these scores range-restricted, so Cohen’s *d* is not interpretable as a cognitive effect size. Age/education-corrected scores were 25.9 ± 0.6 (older) and 28.0 ± 0.5 (younger); the Italian age/education adjustment lowers scores for more-educated participants, which places the more-educated older group below the younger group. The Edinburgh score is strongly ceiling-bound in both groups (36 of 50 older and 18 of 50 younger participants at the maximum), so the parametric comparison reported here understates a rank difference that is significant non-parametrically (Mann–Whitney *p* < 0.001); the robustness analysis restricted to strict right-handers (Supplementary Table S3) shows that this does not affect the primary association.

MRIq and CRIq. Both reserve indices were higher in older adults than in younger adults (MRIq: 62.3 ± 13.0 vs. 48.9 ± 12.9, *d* = 1.04, *p* < 0.001; CRIq: 119.8 ± 15.4 vs. 94.3 ± 6.4, d = 2.15, *p* < 0.001), as expected from the age-cumulative nature of both constructs. Within the older group, MRIq did not correlate with chronological age (*r* = −0.23, *p* = 0.11), so the instrument captured inter-individual variability that age alone does not explain. The within-group correlation between MRIq and CRIq was weak and not significant (older: *r* = −0.20, *p* = 0.16; younger: *r* = +0.19, *p* = 0.19).

### 3.2 Aging effects on motor parameters

Cohen’s d between older and younger adults was computed for each of the 422 informative motor variables (Figures 3, 4). Of these, 42 showed large effects (|d| ≥ 0.8), 96 medium (0.5 ≤ |d| < 0.8), 168 small (0.2 ≤ |d| < 0.5), and 116 negligible (|d| < 0.2). The 12 strongest single effects were concentrated in the externally paced conditions—10 at the metronome and two in continuation—and predominantly on the right hand. Averaged across all variables of a condition, however, the effect is largest at the unimanual metronome (mean |d| = 0.58) and smallest in the two continuation conditions (0.33 unimanual, 0.29 bimanual) (Figure 3). The strongest single-variable effect was the time error of the index finger at the metronome (*d* = +1.68). With our convention that positive TE values denote a delay relative to the metronome onset and negative values denote an advance, younger adults showed the negative mean asynchrony classically reported in sensorimotor synchronization (Repp, 2005; Repp and Su, 2013), tapping markedly ahead of the beat (mean TE ≈ −82 ms; approximately 85% of taps in advance). Older adults, in contrast, tapped close to or slightly after the beat (mean TE ≈ +45 ms; about 57% in advance). The two groups therefore differed in the sign of the time error and not only in its magnitude. Other prominent effects included the time-error indices in the metronome task (*d* ≈ ±1.36), the standard error of touch duration in the continuation task (*d* = −1.45), the metronome hand rate (*d* = +1.21), and the metronome inter-tapping interval (*d* = −1.13). Self-paced and maximum-velocity parameters showed smaller mean effects: Motor performance is more preserved when pacing is unconstrained.

**Figure 3:**
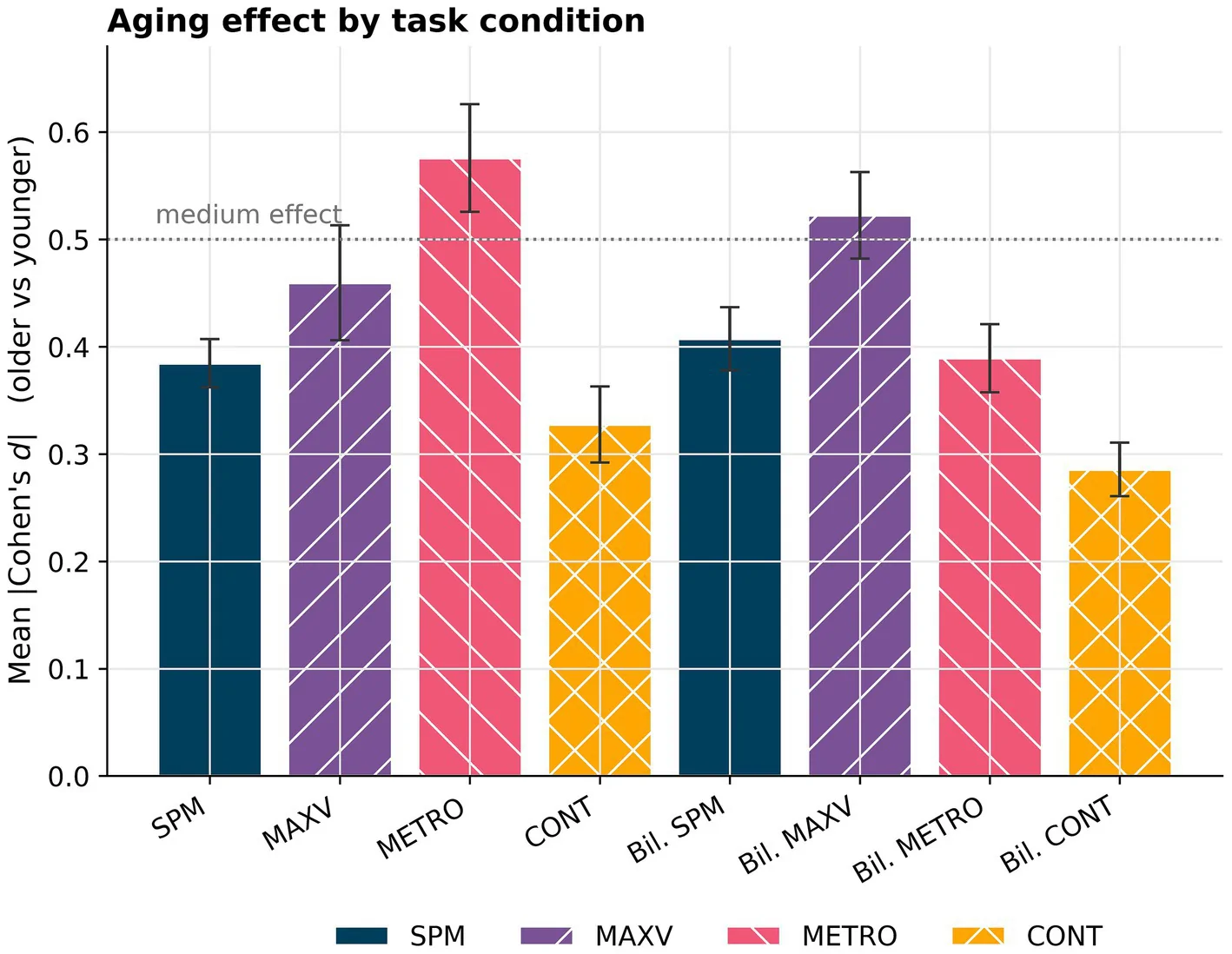
Aging effect by task condition. Mean absolute effect size (|Cohen’s d|, older vs. younger) across the 422 informative motor variables, grouped by task condition; error bars are standard errors. The eight bars correspond to the four tasks in their unimanual and bimanual versions; bars are colored and hatched so that they remain distinguishable in grayscale. The largest mean effect is at the unimanual metronome (0.58), followed by bimanual maximum velocity (0.52); the two continuation conditions are the smallest (0.33 unimanual and 0.29 bimanual).

**Figure 4:**
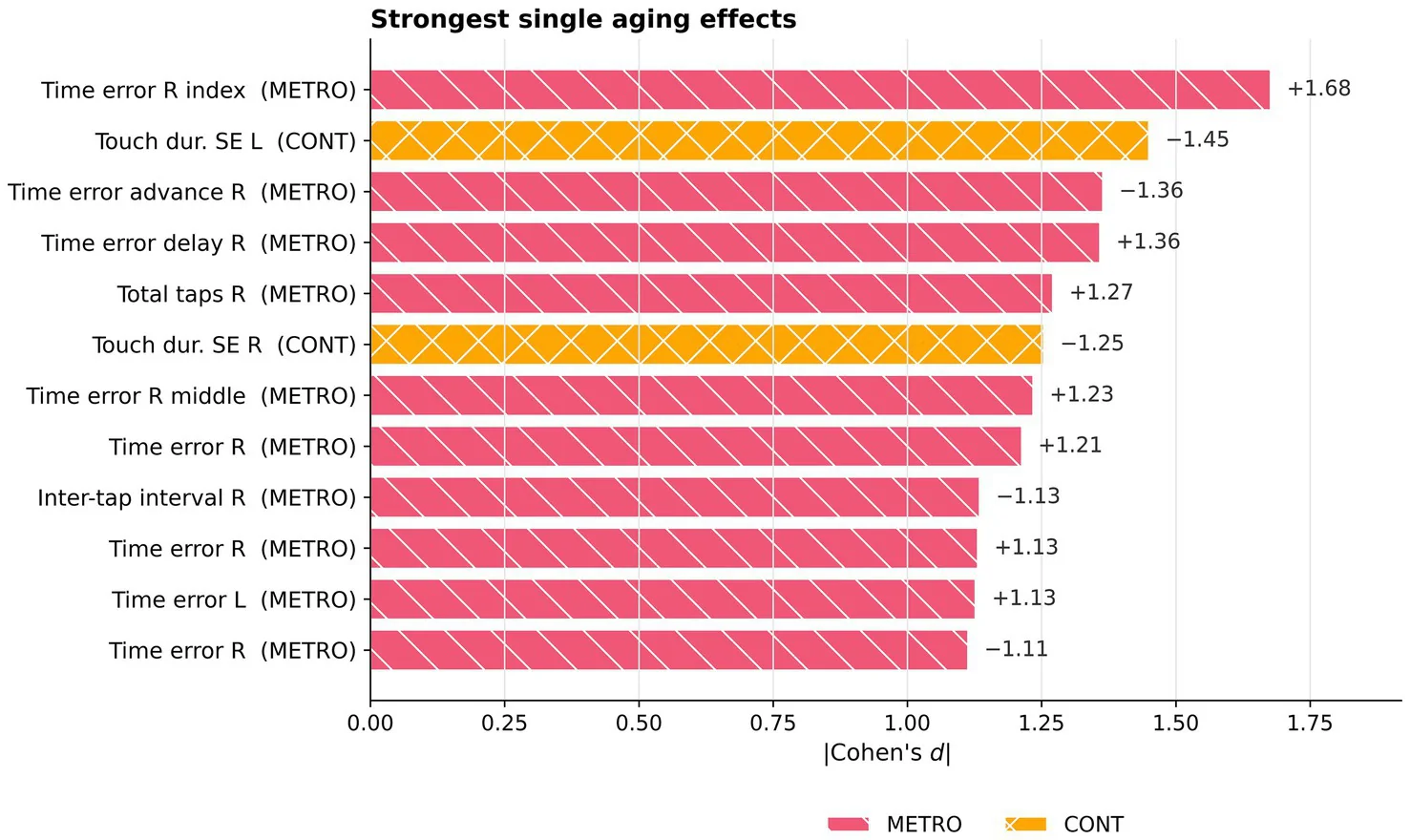
Strongest single aging effects. The 12 individual motor variables with the largest older-vs.-younger effect sizes; the signed Cohen’s d is annotated beside each bar. The strongest single effect is the right index-finger time error at the metronome (*d* = +1.68). Color and hatching indicate task condition.

### 3.3 Pre-specified candidate axes and the confounding by age

This section reports the primary analysis. Its central result is that three of the five candidate axes—A1, endogenous timing stability; A4, touch-duration lateralization; and A5, cross-context consistency—retained an association with MRIq once chronological age was accounted for, whereas A2 and A3 did not. The statistical procedures are described in §2.6.4 and the diagnostic categories in §2.6.3; the results are reported below in the order pooled sample, age-confounding diagnostic, within-group analysis. Because the five axes were selected by their association with MRIq in the older subsample (Supplementary material 1), the within-older association is an internal (in-sample) estimate, bias-corrected by the nested cross-validation (§3.5); the younger group, the group-by-axis interaction, and the discriminant analyses were specified after the axes were locked and therefore constitute prospective tests.

- Pooled analysis (*n* = 100). Four of the five candidate axes survived FDR correction in the pooled sample with the predicted sign: A2, externally paced velocity (*r* = +0.39, pFDR = 2 × 10^−4^); A5, cross-context consistency (*r* = −0.29, pFDR = 0.005); A4, TD lateralization (*r* = −0.24, pFDR = 0.013); and A3, bimanual coordination (*r* = +0.23, pFDR = 0.013), whose positive association matched the direction observed in the exploratory analysis. A1, endogenous timing stability (*r* = −0.14, pFDR = 0.084) did not survive correction. The pooled estimates, however, mix within-group variability with between-group covariation: Both MRIq and most motor indices differ between younger and older adults due to age, which can generate spurious pooled correlations. We therefore moved to a formal diagnosis of confounding.
- Simpson check and within-group analysis. Table 3 reports, for each axis, the within-group Pearson correlations (older and younger), the partial correlation with MRIq controlling for age, and the GROUP × axis interaction in the model MRIq ~ axis + GROUP + axis: GROUP. The diagnoses cluster the five axes in three categories.
- Interpretation. Three axes (A1, A4, and A5) survive both testing within the older group and partial correlation for age: Their effect on MRIq is not absorbed by aging. A4 and A5 also show a significant GROUP × axis interaction, marking them as specific to the older group (a partial Simpson configuration, in which the same-direction effect appears in pooled estimates but diverges between groups). A2, the velocity index at the metronome, has its strongest pooled effect (*r* = +0.39), loses significance within the older group (*r* = +0.19, *p* = 0.18), and barely survives partial correlation (*r* = +0.23). A2 therefore meets the operational definition we use throughout for an aging-driven marker: associated with MRIq in the pooled sample but not within either group, and with no group interaction; the partial correlation, which removes only the linear age term, is the one diagnostic it still passes. A3, bimanual coordination, shows the same pattern as A2: a pooled positive correlation in the predicted direction (*r* = +0.23, pFDR = 0.013) that loses significance within the older group (*r* = +0.16, *p* = 0.28) and under partial correlation for age (*r* = +0.14). A3 therefore meets the same operational definition of an aging-driven marker; its pooled positive sign is carried by the bilateral inter-tap-interval variability, which increases with age in parallel with the age-cumulative MRIq.

**Table 3:** Diagnosis of confounding by age for the five candidate axes.

Axis	r all (*n* = 100)	r old (*n* = 50)	r young (*n* = 50)	r partial (Age)	p interaction	Diagnosis
A1—Timing endogenous	−0.14	−0.37**	−0.10	−0.23*	0.44	Genuine, suppressed by pooling
A2—Velocity (metro)	+0.39***	+0.19	+0.14	+0.23*	0.81	Pooled-only, age-driven
A3—Bimanual coordination	+0.23*	+0.16	+0.08	+0.14	0.92	Pooled-only, age-driven
A4—TD lateralization	−0.24*	−0.49***	+0.08	−0.23*	0.005	Partial Simpson, specific to older group
A5—Cross-context IIV	−0.29**	−0.40**	+0.08	−0.18*	0.014	Partial Simpson, specific to older group

Significance markers refer to one-tailed *p*-values for partial correlations (consistent with the directional pre-specification of the axis sign) and two-tailed *p*-values for the GROUP × axis interaction. *p < 0.05; **p < 0.01; ***p < 0.001 (FDR-corrected on *n* = 100 for partial correlations; uncorrected p for within-group).

### 3.4 Evidence weight: Bayes factors

To distinguish absence of evidence from evidence of absence on non-significant axes, we computed Bayes factors (BF₁₀) for each candidate axis × MRIq within the older subsample (Table 4). BF₁₀ ranged from decisive evidence for H1 (rMR BF₁₀ ≈ 2,080; A4 BF₁₀ ≈ 119) to moderate evidence for H0 (A3 BF₁₀ = 0.26). A1 and A5 produced moderate-to-strong evidence for H1 (BF₁₀ = 6.10 and 10.2). A2 and A3 both fell in the weak range (BF₁₀ = 0.36 and 0.26). These two values lie on either side of the conventional 1/3 boundary but are not meaningfully different from each other, and we do not treat them as qualitatively distinct: For both axes, the older subsample provides only weak evidence against a within-group association with MRIq, marginally more so for A3. Neither value licenses a positive claim that the effect is absent. Because rMR and the retained axes were selected on the same older subsample, these Bayes factors quantify in-sample evidence and are not independent confirmation; the nested cross-validation in §3.5 provides the out-of-sample counterpart.

**Table 4:** Bayes factors (BIC approximation) for each candidate axis against MRIq within the older subsample (*n* = 50).

Axis	r within the older group	BF₁₀ (bidirectional)	Interpretation (Jeffreys)
rMR (composite)	+0.57	≈ 2,080	Decisive for H1
A4—Touch-duration lateralization	−0.49	≈ 119	Decisive for H1
A5—Cross-context consistency	−0.40	≈ 10.2	Strong for H1
A1—Endogenous timing stability	−0.37	≈ 6.10	Moderate for H1
A2—Externally paced velocity	+0.19	≈ 0.36	Anecdotal for H0
A3—Bimanual coordination	+0.16	≈ 0.26	Moderate for H0

BF₁₀ is the ratio of the evidence for the alternative hypothesis over the null. The values for A2 and A3 lie either side of the conventional 1/3 boundary but are not meaningfully different from each other (§3.4). Because the axes were selected on this same subsample, these Bayes factors quantify in-sample evidence.

### 3.5 Multivariate predictive models within the older group

Three multivariate models for MRIq were estimated within the older subsample (Table 5). The full model with all five candidate axes yielded *R*^2^ = 0.37 in-sample and R^2^LOO = 0.19; the reduced model with the three robust axes (A1, A4, and A5) yielded *R*^2^ = 0.34 and R^2^LOO = 0.20; the simple regression on the rMR composite yielded *R*^2^ = 0.32 and R^2^LOO = 0.25 (Figure 5). The single-predictor rMR model achieved the numerically best (though statistically indistinguishable from the multivariate models) out-of-sample performance: leave-one-out cross-validated *r* = +0.50 between predicted and observed MRIq, pperm < 0.001 (1,000 permutations). When the data-driven selection of the three robust axes was nested within the cross-validation, out-of-sample performance was attenuated but remained significant (R^2^LOO = 0.16, *r* = +0.42, permutation *p* = 0.001). In 49 of the 50 folds, the nested selection returns the same three axes and the same predictions; the whole of the attenuation comes from the single fold that holds out the high-leverage participant described in §3.7. The nested estimate and the high-leverage-excluded estimate therefore bear on that one observation rather than on two independent sources of uncertainty. The bivariate Pearson correlation between rMR and MRIq was *r* = +0.565 [*p* = 2 × 10^−5^; bootstrap 95% CI (+0.27, +0.76); 99% CI (+0.19, +0.80); 20,000 resamples]. Across the 50 leave-one-subject-out resamples, the bivariate correlation ranged from *r* = 0.42 to *r* = 0.59: No single subject drove the result.

- Group specificity of the residual reserve effect. Two convergent tests, reported here because they qualify the association just described, verify that the predictive effect of rMR on MRIq is specific to the older group.
- Within-young correlation. In the younger subsample, rMR showed essentially no correlation with MRIq (*r* = −0.058, *p* = 0.69). This null result is what a Stern-style residual reserve index should produce: Younger adults are the no-deficit baseline against which the residuals are computed, so they should not exhibit between-individual variation in residual reserve linked to MRIq.
- GROUP × rMR interaction. In a regression on the full sample (*n* = 100), the interaction term GROUP × rMR was significant (βint = −10.83 in unstandardized units, *p* = 0.009): The effect of rMR on MRIq differs between groups.

**Table 5:** Multivariate models for MRIq within the older subsample (*n* = 50).

Model	*N*	*R*^2^ in-sample	*R* ^2^_LOO_	*r*_LOO_	*p*_perm_
Full multivariate (five axes)	50	0.37	0.19	+0.46	–
Reduced multivariate (A1 + A4 + A5)	50	0.34	0.20	+0.46	–
rMR (single predictor)	50	0.32	0.25	+0.50	< 0.001
rMR + AGE	50	–	0.25	+0.50	–

All cross-validated values are leave-one-out. Adding chronological age as a covariate to rMR (last row) does not improve out-of-sample prediction, which is the expected consequence of the residualization on the younger baseline having already absorbed the age signal.

**Figure 5:**
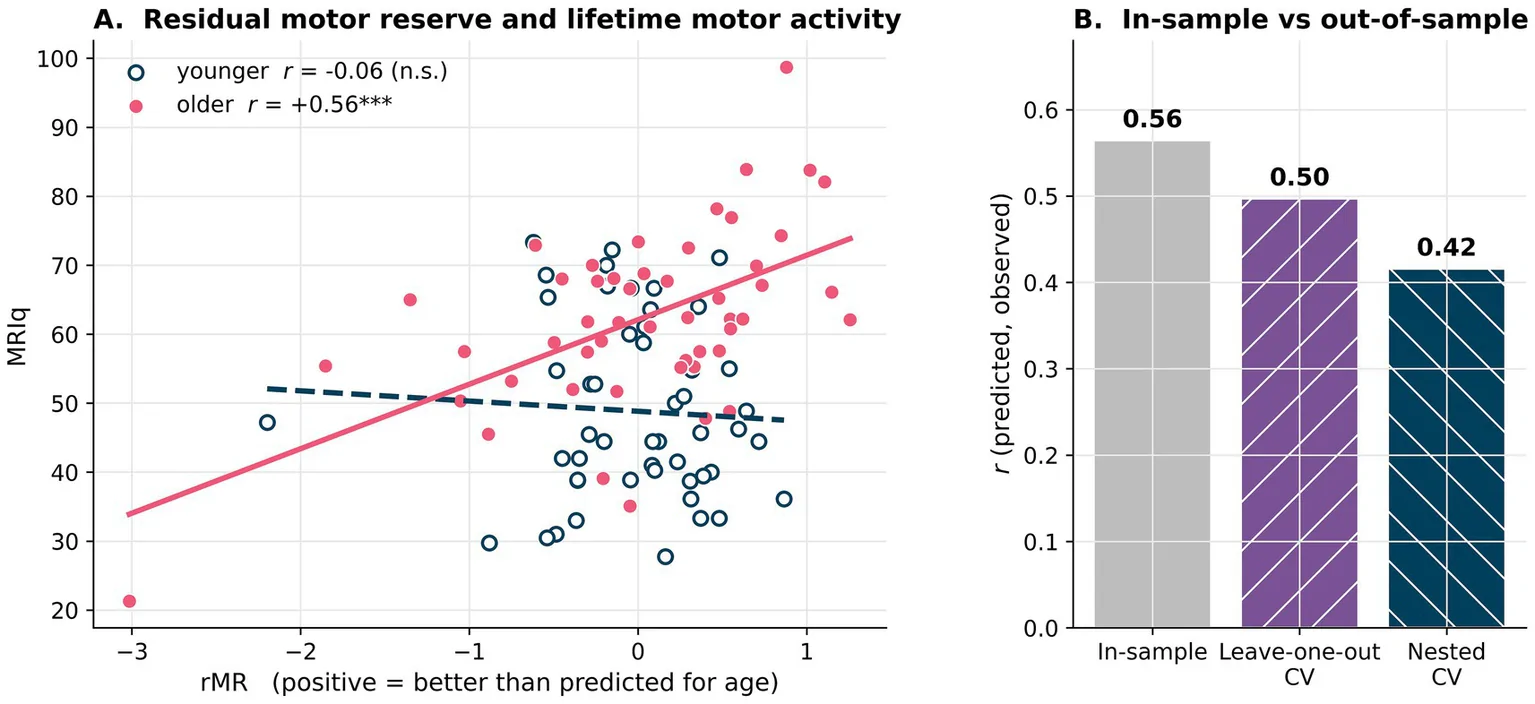
Residual motor reserve and its association with lifetime motor activity. **(A)** Scatter of rMR against MRIq with separate linear fits for the older group (*r* = +0.565, *p* = 2 × 10⁻⁵) and the younger group (*r* = −0.06, n.s.); the association is present only in the older group. **(B)** Predictive performance for MRIq within the older group: in-sample Pearson’s r, leave-one-out cross-validated r, and nested cross-validated r, in which the axis-selection step is repeated inside each fold. The association attenuates from *r* = 0.56 to *r* = 0.42 but remains significant (permutation *p* = 0.001). Influence diagnostics for the two extreme observations are reported in §3.7 and Supplementary Table S3.

### 3.6 Discriminant validity: motor reserve vs. cognitive reserve and cognitive performance

To check that the motor profile captured by rMR is specific to motor reserve rather than a general marker of preserved function, we compared its predictive performance for three parallel outcomes within the older subsample: MRIq, CRIq, and the neuropsychological composite (Table 6). rMR correlated strongly with MRIq (*r* = +0.57) and weakly or not at all with CRIq (*r* = −0.13, *p* = 0.38) and with the neuropsychological composite (*r* = −0.18, *p* = 0.22). The same pattern held for the three component axes A1, A4, and A5 (Table 6).

**Table 6:** Pearson’s correlations of motor reserve predictors with three outcome variables within the older subsample (*n* = 50).

Predictor	→ MRIq	→ CRIq	→ Neuropsychological composite
rMR	+0.57***	−0.13	−0.18
A1—Timing endogenous	−0.37**	+0.03	+0.06
A4—TD lateralization	−0.49***	+0.06	+0.12
A5—Cross-context IIV	−0.40**	+0.19	+0.24

**p < 0.01 and ***p < 0.001.

Differences between dependent correlations were tested with Steiger’s z in the predictor-shared form (Williams, 1959; Steiger, 1980). For rMR, the correlation with MRIq was significantly stronger than the correlation with CRIq (*z* = +3.31, *p* < 0.001) and with the neuropsychological composite (*z* = +3.72, *p* < 0.001) (Figure 6). The correlations with CRIq and the neuropsychological composite did not differ from each other (*z* = +0.26, *p* = 0.80), unsurprisingly since both were near zero. See Supplementary material for further details about this analysis.

**Figure 6:**
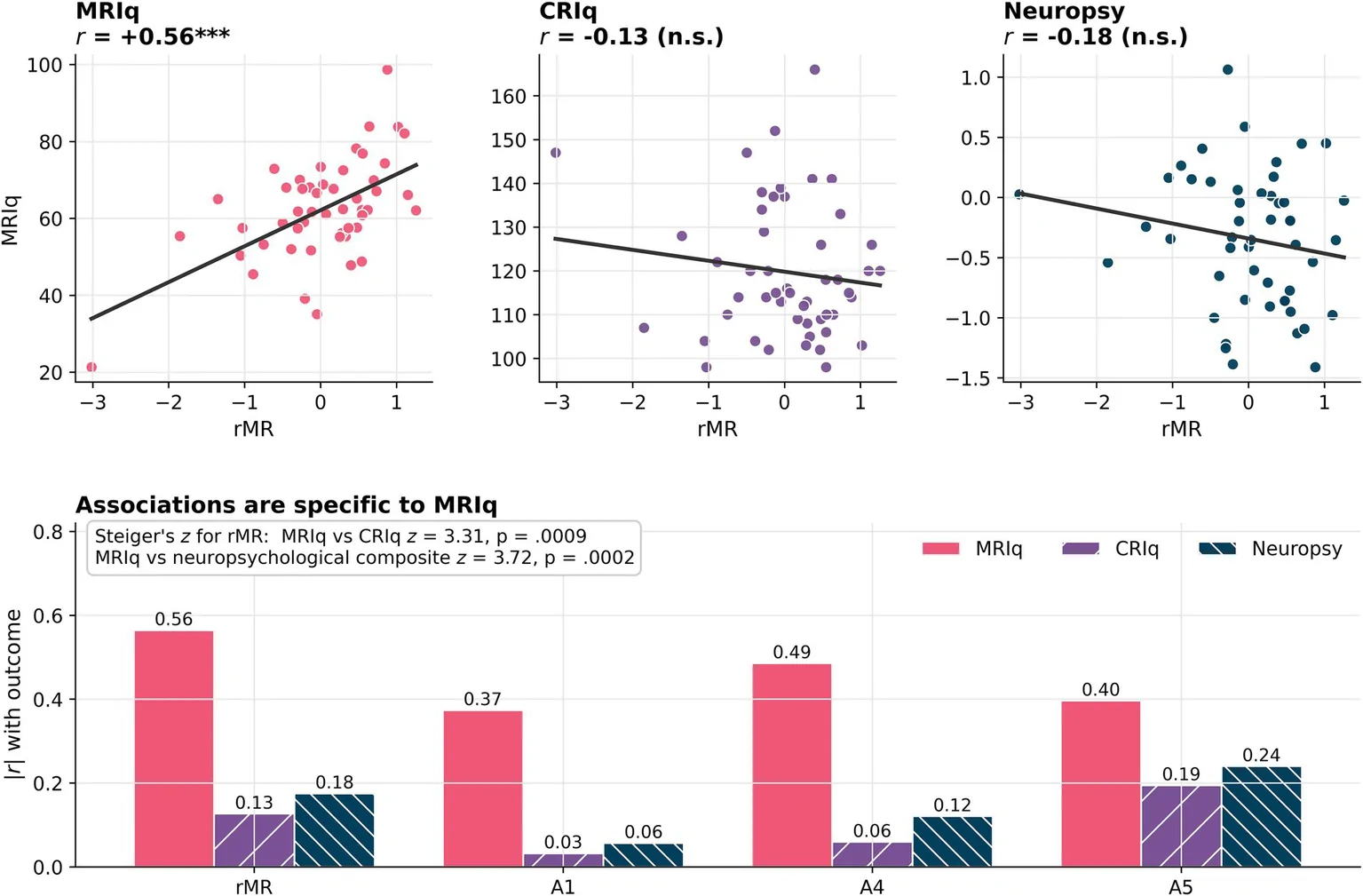
Discriminant validity of the residual reserve index (older subsample, *n* = 50). (Top) Scatter plots of rMR against MRIq, CRIq, and the neuropsychological composite, with within-group correlations. (Bottom) Absolute correlations of rMR and its component axes (A1, A4, and A5) with the three outcomes; the association is specific to MRIq. Steiger’s *z*-tests confirm a stronger correlation of rMR with MRIq than with CRIq (*z* = 3.31, *p* < 0.001) or the neuropsychological composite (*z* = 3.72, *p* < 0.001).

### 3.7 Sensitivity and robustness analyses

Robustness was assessed with the analyses specified in §2.6.8; full results are reported in Supplementary Tables S3, S4. Two points belong in the main text. First, what does not change: the direction and significance of the rMR × MRIq association within the older group are preserved under every specification examined—restriction to strict right-handers (*r* = +0.57), exclusion of multivariate outliers, adjustment for sex, education and age, rank-based and log-transformed correlations, robust regression, and ordinal treatment of MRIq. Second, what does change: the magnitude of the Pearson coefficient is sensitive to a single high-leverage observation, falling from *r* = +0.565 to *r* = +0.421 when that participant is excluded, whereas the rank-based estimate barely moves (ρ = +0.46 to +0.43). The point estimate should therefore be read as an interval rather than as a value, with the Spearman correlation and the bounded-influence estimator (βstd = +0.54) as the more conservative summaries. The association was likewise invariant to the residualization baseline: Robust (Theil–Sen, Huber), quadratic, and intercept-only baselines estimated on the younger group all yielded *r* = 0.56–0.58 (range across the alternative baselines); because the younger-group age slope did not differ from zero, the residualization reduces, within this design, to a standardization of older performance. The within-older association was also unchanged when MRIq was itself residualized on age, or when age was partialed from both variables (*r* = +0.56 in both cases), confirming that it is not carried by the age-cumulative nature of MRIq, which within the older range did not increase with age (§3.1). A complete description and discussion of all sensitivity analyses and their results is provided in Supplementary material.

### 3.8 Finger-level decomposition of the lateralization axis

Because A4 is the dominant component of the composite (§3.5), we decomposed it by finger and by task condition. Within the older group, the association with MRIq was not distributed uniformly across effectors: It was carried by the middle finger and by the four-finger mean under the two most demanding pacing conditions (middle finger, maximum velocity *r* = −0.42, *p* = 0.002; middle finger, continuation *r* = −0.40, *p* = 0.004; four-finger mean, *r* = −0.40 and *r* = −0.35 in the same two conditions). The ring finger contributed at intermediate strength in both conditions (*r* ≈ −0.35) and the little finger only under continuation (*r* = −0.34), while the index finger contributed weakly. The self-paced and metronome conditions produced no significant finger-level associations. The full condition × finger matrix is given in Figure 7. This analysis was exploratory, and its correlations are computed within the subsample on which A4 was defined; a possible interpretation in terms of finger individuation and lateralized motor organization is considered in §4.3.

**Figure 7:**
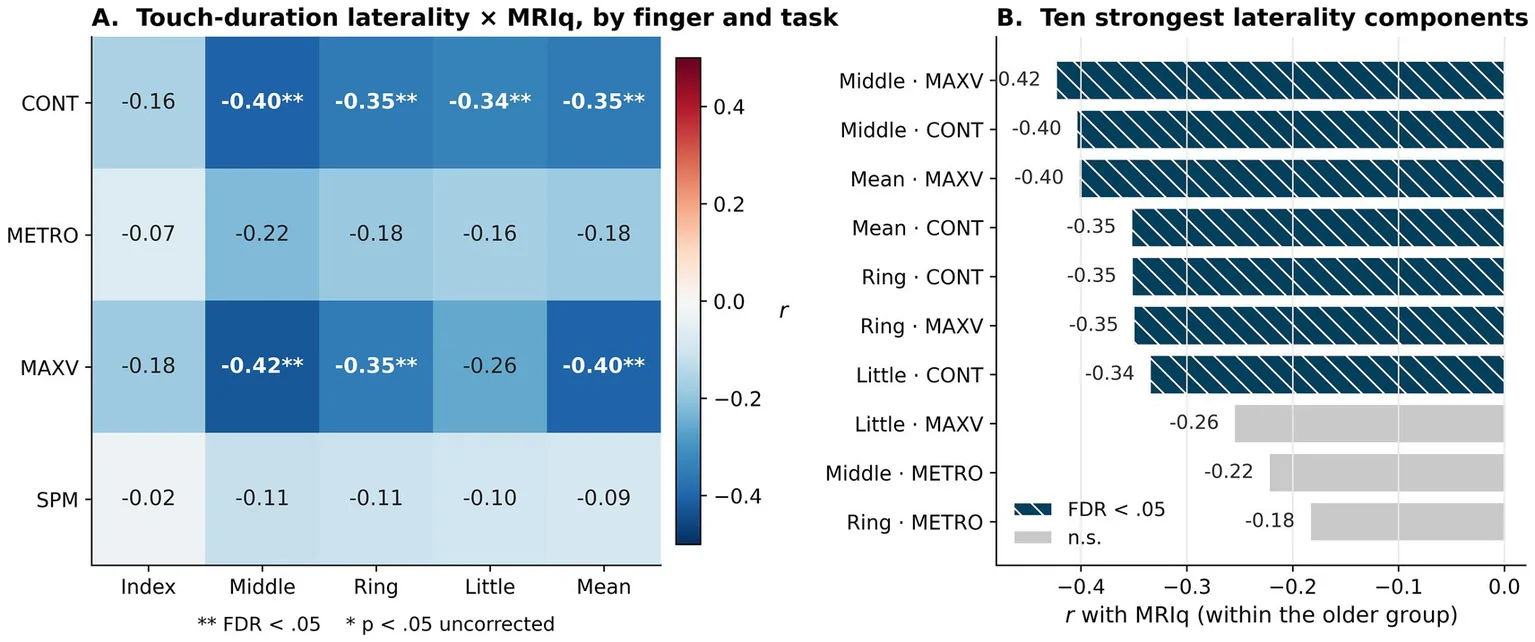
Finger-level decomposition of the touch-duration lateralization axis (A4), older group, *n* = 50. **(A)** Pearson’s correlations between the laterality index of touch duration and MRIq for each finger and each task condition; **marks associations surviving FDR correction, *uncorrected *p* < 0.05. **(B)** The 10 strongest components, ordered by magnitude. The association is concentrated on the middle finger and the four-finger mean under maximum-velocity and continuation pacing and is absent under self-paced and metronome-paced execution. These correlations are computed within the subsample on which A4 was defined and are therefore in-sample; the analysis is exploratory and is interpreted in §4.3.

### 3.9 Age-independence of the residual index within the older group

Within the older subsample (age range 50–76 years), rMR did not correlate with chronological age (*r* = −0.09, *p* = 0.54). The same holds for each component axis: A1 (*r* = −0.04, *p* = 0.76), A4 (*r* = +0.15, *p* = 0.29), and A5 (*r* = +0.05, *p* = 0.74). MRIq itself showed only a weak non-significant decline (*r* = −0.23, *p* = 0.11). Within the sampled older range, rMR therefore separates participants with more motor reserve from those with less independently of their chronological age, without further linear age regression. This absence of a within-group age gradient is in part a constructive consequence of residualizing each axis against an age model rather than independent evidence of trait-like stability, and we do not interpret it as such; its implications and limits are discussed in §4.4.

## 4 Discussion

### 4.1 Summary of findings

A central question in the study of motor aging is why some individuals maintain efficient motor performance despite widespread age-related changes, while others decline more markedly. In this study, we addressed this issue by asking whether motor reserve—traditionally inferred from life-history measures—can be captured objectively through fine-grained kinematic parameters, and, critically, whether it can be distinguished from the broader effects of chronological aging itself. These two questions are inseparable, for the reason developed in §4.2: a measure that simply tracks how old someone is, however precise, is uninformative about reserve. To examine this distinction, we defined five candidate composite indices reflecting complementary dimensions of motor behavior: endogenous timing stability (A1), which captures the regularity of internally generated finger movements; externally paced velocity (A2), which reflects tapping speed under metronome guidance; bimanual coordination (A3), which indexes the stability of coupling between the two hands; touch-duration lateralization (A4), a right–left asymmetry index used here as a proxy for the dominant–non-dominant contrast; and cross-context consistency (A5), which reflects how reliably an individual reproduces a motor profile across different task demands. However, once the confounding influence of chronological age was formally considered, this initial picture became more selective and more informative. Externally paced velocity (A2), despite showing one of the strongest pooled effects, emerged as a prototypical aging-driven marker: It primarily reflected how old someone was, rather than how much reserve they had. Bimanual coordination (A3) did not provide reliable evidence of a reserve-related association. In contrast, endogenous timing stability (A1), touch-duration lateralization (A4), and cross-context consistency (A5) remained associated with MRIq beyond age effects, indicating that motor reserve is not captured by faster movement alone, but as a more stable, lateralized, and consistent organization of motor behavior. In simple terms, chronological age mainly tells us about tapping velocity at the metronome, whereas motor reserve tells us something different: how stable internally generated timing remains, how much the dominant hand continues to lead under demanding conditions, and how consistently motor performance is maintained across changing contexts. Thus, lower velocity indexes aging, whereas lower variability and more preserved lateralization index reserve.

To quantify this effect, we adopted a Stern-inspired residual approach and combined the three robust axes into a residual motor reserve index, quantifying the extent to which each older participant performed better than expected for age relative to a younger no-deficit baseline. This residual index strongly predicted MRIq within older adults, showed the best out-of-sample predictive performance, and was not associated with MRIq in younger participants, with a significant group-specific effect. This pattern indicates that the association between lifetime motor activity and the kinematic profile identified here is detectable in older adults but not in the younger baseline group. Given the age-cumulative nature of MRIq, the younger participants serve primarily as a kinematic reference rather than as a comparison group for motor reserve itself. Overall, these findings suggest that motor reserve should not be understood as the simple preservation of speed but as the preservation of an organized motor phenotype: stable in its timing, lateralized in its execution, and consistent across changing task demands.

Three additional findings delimit our new construct of motor reserve: (a) Discriminant validity. The residual reserve index correlated strongly with MRIq, but not with the cognitive reserve index (CRIq, *r* = −0.13) or with a composite of cognitive performance (*r* = −0.18); Steiger’s z confirmed that the differences between dependent correlations were significant (z > 3.3, *p* < 0.001 in both contrasts). (b) Within-older age-independence. Within the older subsample (50–76 years), rMR did not correlate with chronological age (*r* = −0.09, *p* = 0.54): Inter-individual variation in residual reserve dominates over any further linear age regression in this window (the structural and longitudinal interpretation is detailed in §4.4). (c) Anatomical specificity. Jackknife analysis identified A4 (TD lateralization) as the dominant component; finger-by-finger dissection localized the effect on the middle finger and its neighbors in the maximum-velocity and continuation tasks, i.e., the most demanding pacing conditions on the fingers with the most differentiated cortical representation. Our findings can be interpreted within a broader framework in which motor reserve reflects not the preservation of absolute motor output but the maintenance of adaptive motor capacity. Recent evidence from multimodal behavioral and neuroimaging studies (Kögler et al., 2025) shows that while gross motor performance declines with age, motor adaptation abilities remain relatively preserved across the lifespan and are closely linked to the integrity of motor networks and lifelong physical activity experience. This distinction provides a critical conceptual anchor: Motor reserve does not manifest as faster or stronger performance per se but as the ability to flexibly maintain stable and efficient motor behavior despite aging-related constraints.

### 4.2 Separating aging from reserve

The first question, whether sensor-equipped glove parameters yield objective biomarkers of motor reserve, cannot be addressed without first solving the second: how to separate the contribution of motor reserve from the much broader contribution of chronological aging. In our sample, 138 of 422 motor variables (33%) showed medium-to-large age effects (|d| ≥ 0.5) and 42 (10%) showed large effects (|d| ≥ 0.8). These include core measures of motor performance: tapping rate at the metronome, time error, inter-tapping intervals, and counts of correctly executed sequences. In a bimodal sample where age and group are quasi-aliases, the pooled correlation between any age-sensitive motor variable and any age-sensitive measure (MRIq is a cumulative life-history score, hence age-sensitive) will be inflated by the joint covariation of both with age. Pooled significance under FDR correction is therefore a necessary but not sufficient criterion for an axis to qualify as a biomarker of motor reserve.

Motor reserve, adopting the Stern-inspired framework (Stern, 2002, 2009; Cabeza et al., 2018; Stern et al., 2020), is the residual variability in functional capacity that cannot be attributed to chronological aging or its biological correlates: The component that distinguishes individuals aging better than age alone would predict. A candidate biomarker that varies with age but does not vary among older adults of the same age captures aging, not reserve. This is what happened in our sample for the externally paced velocity index (A2): the strongest pooled correlation with MRIq (*r* = +0.39), vanishing within the older subsample (*r* = +0.19, *p* = 0.18), with an inconclusive Bayes factor (BF₁₀ = 0.36). A2 is best read as a marker of preserved motor capacity: informative about the difference between younger and older adults and indirectly correlated with MRIq through the shared latent age dimension, but uninformative about motor reserve in the strict sense.

Three of our candidate axes passed the age-confounding diagnostic: A1 (endogenous timing stability), A4 (TD lateralization), and A5 (cross-context consistency). Each shows a significant within-group correlation with MRIq, a robust partial correlation controlling for age, and (for A4 and A5) a significant GROUP × axis interaction that confirms the effect emerges specifically in the older group. The rMR composite, constructed by regressing these three axes on age in the younger group and computing residuals for the older group, formalizes the distinction. A positive rMR value for an older participant means that the participant performed better than the value extrapolated from the younger-adult baseline at their age, i.e., motor function exceeds the no-deficit projection net of the average aging effect. The strong association of this residual with MRIq within the older group (*r* = +0.57) and its null association with MRIq within the younger group (*r* = −0.06) are the two properties expected of a strict-sense motor reserve index.

### 4.3 Residual motor reserve index: a new three-dimensional construct

The three axes that passed the age-confounding diagnostic identify three distinct dimensions of motor performance whose preservation maps onto greater motor reserve.

- Endogenous timing stability (A1). Operationalized as the standard deviation of right-hand touch duration in the self-paced task, this axis indexes the temporal regularity of finger-to-thumb contact when no external pacer is available. The negative within-older-group association with MRIq (*r* = −0.37; moderate-to-strong Bayes factor, BF₁₀ = 6.10) means that older participants with greater motor reserve show less trial-to-trial variability in touch duration. The result aligns with the literature on intra-individual variability as a marker of neural integrity (Hultsch and MacDonald, 2004; MacDonald and Meck, 2004), where reduced variability indexes more efficient and better-tuned cortico-motor circuits.
- TD lateralization (A4). A4 is the dominant component of the residual reserve composite: Removing it collapses the three-axis model from R^2^LOO = 0.20–0.06, whereas removing A1 or A5 leaves it essentially unchanged (0.20 and 0.19). The axis quantifies the asymmetry of touch duration between dominant and non-dominant hands under demanding pacing (maximum velocity and continuation). The dissection analysis (§3.8) localized the effect on the middle finger and on the four-finger mean, with progressively weaker contributions from the ring and little fingers and essentially no contribution from the index finger or from the self-paced and metronome conditions. The anatomical pattern fits known properties of finger individuation: the middle finger sits at the center of the four-finger somatotopy, and quantitative mapping of finger independence (Häger-Ross and Schieber, 2000) shows that the ring and little fingers are mechanically and neurally constrained to it, while the index escapes much of this coupling. The motor representations themselves are highly distributed and overlapping in the primary motor cortex (Schieber, 2001; Indovina and Sanes, 2001), so the asymmetry between dominant and non-dominant hands becomes most discriminative on the central finger of the chain when the system is taxed. The functional consequence is a natural stress test of the lateralized motor architecture: precisely because the middle finger is so deeply coupled to its neighbors, tapping it independently and rapidly under maximum-velocity or continuation demand requires the cortico-motor system to actively inhibit the adjacent fingers. On this account, the harder the inhibitory demand, the more it would discriminate between individuals whose lateralized motor circuits are better or less well preserved, and older adults with greater motor reserve would be those able to sustain that demand more efficiently, maintaining a greater dominant-vs.-non-dominant asymmetry under load. We stress that this is an interpretation of a behavioral pattern and not a demonstrated mechanism: The present study measures movement only and provides no measure of cortical lateralization, interhemispheric inhibition, or the structural integrity of motor pathways. Testing it would require combining this protocol with neurophysiological or imaging measures. Maximum velocity and continuation impose more demanding constraints on the lateralized motor system than self-paced or metronome-paced execution, which can rely on a global pacing template distributed more symmetrically across hemispheres. The negative within-older-group association with MRIq means that older adults with greater motor reserve show more pronounced lateralization: The dominant hand maintains shorter touch durations than the non-dominant hand to a greater extent under load. This effect is directional: An unsigned laterality magnitude does not reproduce it (within the older group, *r* = +0.26), confirming that what tracks motor reserve is the dominant-hand advantage specifically, not the size of the asymmetry. The pattern fits the preservation of lateralized motor architecture as a marker of reserve and parallels reports of reduced hemispheric specialization in less-reserved aging brains (Cabeza, 2002; Park and Reuter-Lorenz, 2009).
- Cross-context consistency (A5). A5 is the standard deviation across the four task conditions (SPM, MaxV, Metro, and CONT) of basic timing parameters (right-hand TD variability, and right- and left-hand mean ITI). The axis captures the coherence of motor performance when task demands change. Within the older group, lower cross-context variability (a more consistent profile across pacing demands) was associated with higher MRIq (*r* = −0.40, BF₁₀ = 10.2). The result is the within-individual analog of the variability-as-integrity framework and parallels findings in cognitive aging on intra-individual cross-task consistency (Hultsch and MacDonald, 2004). A5 reflects how smoothly the motor system reconfigures across different pacing demands; older participants with greater motor reserve appear to deploy this reconfiguration more uniformly.

In this respect, the present findings converge with the notion that motor reserve is tightly linked to adaptive motor capacity. Motor-perturbation paradigms indicate that adaptation is partly dissociable from raw movement speed and is not uniformly degraded by aging: Older adults retain visuomotor adaptation and its aftereffects (Cressman et al., 2010; Lazar-Kurz et al., 2023) and adapt to split-belt locomotion at rates comparable to younger adults (Malone and Bastian, 2015; Kuhman et al., 2022), whereas the more strategic and retention-dependent components decline with age (Simon and Bock, 2015; Malone and Bastian, 2015) and adaptive control of balance changes across the lifespan (Vervoort et al., 2020). This literature concerns chronological age rather than reserve, and we are not aware of perturbation studies that stratify participants by motor reserve. The reduced intra-individual variability captured by A1 and A5 is therefore best described as a potential kinematic correlate of adaptive capacity: a hypothesis that the present cross-sectional design, which includes no adaptation or perturbation task, motivates but cannot test (Kögler et al., 2025).

### 4.4 Limitations

This study is characterized by some specific limitations, listed below. The distinction that governs all of them is between internal robustness and external validation: The analyses reported above establish that the association we describe is not an artifact of a particular analytical choice, but none of them establishes that it generalizes beyond this sample.

#### 4.4.1 Sample size, age range, and cross-sectional design

The older subsample (*n* = 50) gives reasonable but not large statistical power, so the bootstrap 95% confidence interval for the rMR × MRIq association [CI (+0.26, +0.76)] is broad. The bimodal design also means that age and group are confounded by construction: The two are near-aliases, which is why the age-confounding diagnostic is central to our analysis rather than an accessory check. The residual approach mitigates this confounding but cannot substitute for continuous sampling across the adult lifespan, and the generalizability of the index should be judged accordingly. The design also leaves two parts of the lifespan unsampled. The intermediate range (35–50 years) is sparse, so the rate at which residual reserve emerges between young adulthood and middle age cannot be characterized here. The very-old range (>76 years) is also absent: The upper edge of our older sample stops below the age window in which most age-related motor decline accelerates and in which a reserve buffer would be expected to matter most. Generalization to either the middle or the late-old portion of the lifespan therefore requires future studies with continuous age sampling across the adult lifespan, including a substantial number of participants beyond 75 years. The study is also strictly cross-sectional. The absence of an age gradient for rMR within the older group is consistent with, but does not demonstrate, longitudinal stability of the index and is in any case partly a corollary of the residualization itself. Longitudinal designs are needed to test whether rMR tracks individual trajectories of preserved motor function across years and whether baseline rMR predicts subsequent functional change.

#### 4.4.2 Sensitivity to the extreme tail of the distribution

One participant (ID29) is a high-leverage observation whose exclusion moves the Pearson coefficient substantially while leaving the rank-based estimate almost unchanged (§3.7). Excluding this participant lowers the Pearson coefficient from *r* = +0.565 to *r* = +0.421, while the Spearman coefficient moves from ρ = +0.46 to +0.43 and the bounded-influence estimate is βstd = +0.54. The association therefore holds with and without the observation, but at *n* = 50 its magnitude is not precisely estimated: We report it as the interval spanned by these estimates rather than as a point value, and comparisons with future studies should be made on the rank-based or bounded-influence estimators. The case was retained because its studentized residual places it on the regression line rather than off it—it is an extreme value of MRIq, not a discrepant one. The direction, the group specificity, and the discriminant pattern are unaffected either way.

#### 4.4.3 Pre-specification, not pre-registration

The five candidate axes, their predicted signs, and the age-confounding reduction rule were fixed on the older-subsample exploration and locked before the younger group was collected but were not deposited in a public registry. Only the within-older association is therefore in-sample, and its nested cross-validated value (§3.5) is the bias-corrected estimate; the younger-group null, the group-by-axis interaction, the residualization baseline, and the discriminant analyses are prospective. Within the older subsample, the three retained axes remain significant under two-tailed tests (A1, *r* = −0.37; A4, *r* = −0.49; A5, *r* = −0.40; all *p* ≤ 0.008, *n* = 50). One asymmetry bounds the cross-group comparison: MRIq accumulates with lifetime exposure, so a participant in their twenties cannot score highly whatever their reserve. The younger group is a kinematic baseline and not a reserve comparison group, and the within-younger null (*r* = −0.06) is a negative control on the kinematic side rather than evidence that reserve is absent in young adults. Replication should register these axes externally and test them in an independent sample.

#### 4.4.4 Item-level MRIq analysis not available

The dataset includes only the MRIq total score, not the individual items or sub-scales. We could not test whether rMR correlates differentially with sub-components of self-reported motor reserve (sport activities, occupational manual demands, and hobbies). The question is relevant for clarifying which life-history experiences contribute most to the objective motor profile, and we plan to address it in follow-up work where MRIq item-level data are available.

#### 4.4.5 External replication

The composite indices were selected on the older subsample (*n* = 50) (Supplementary material 1) and validated on the full dataset (*n* = 100). The within-older-group result on *n* = 50 is methodologically a reanalysis of the subset on which the index was constructed. Consistent with this, nesting the axis-selection step within the cross-validation reduced out-of-sample performance from R^2^LOO = 0.25–0.16 (*r* = +0.42), although the association remained significant (pperm = 0.001). As noted in §3.5, that reduction is carried by 1-fold rather than by instability of the selection itself, and it is not independent of the influential-observation analysis. The sensitivity and Bayesian analyses, together with the null finding in the younger baseline, therefore, speak to internal robustness and not to generalization, which this design cannot address.

Within the measures available here, rMR tracked the motor-exposure questionnaire and not the cognitive reserve questionnaire or cognitive performance. We read this as evidence of relative specificity rather than of independence: A dissociation observed in one sample, with a single instrument per construct, cannot establish whether motor and cognitive reserve are separate constructs or components of one higher-order construct. It does support the weaker claim that motor reserve is not a redundant relabeling of generic successful aging.

### 4.5 Toward neural validation

The present study characterizes motor reserve at the behavioral level only. Anchoring rMR in identifiable neural substrates is the natural next step and would also arbitrate between the interpretations offered above. Combining the residual reserve approach with structural and functional neuroimaging—white-matter microstructure of motor tracts (Oschwald et al., 2021), cerebellar volumetric measures (Arleo et al., 2023), or task-related functional MRI of lateralized motor networks—would test whether the three retained axes map onto dissociable neural systems, and whether the lateralization effect in particular reflects preserved interhemispheric organization. We frame this as a direction for future validation rather than as an extension supported by the present data.

## 5 Conclusion

This study addressed a fundamental question: can motor reserve be measured objectively, and can it be distinguished from the broader effects of aging? Our findings show that answering the second question is essential to answering the first. Many motor features change with age, but only some capture the individual capacity to maintain performance despite aging. When we accounted for age-related effects, three specific dimensions of motor behavior emerged as reliable markers of motor reserve: endogenous timing stability (A1), touch-duration lateralization between the hands (A4), and cross-context consistency across tasks (A5). These indices describe how stable, well-organized, and consistent motor performance is across different conditions. In contrast, externally paced velocity (A2), which reflects tapping speed under metronome guidance, primarily indexed chronological age rather than motor reserve, while bimanual coordination (A3) did not show a consistent relationship with reserve. By combining the three robust dimensions (A1, A4, and A5), we derived a composite residual motor reserve index (rMR) that captures how much an individual performs better than expected for their age, relative to a younger baseline. This index was strongly associated with lifetime motor activity (MRIq) in older adults, but not in younger individuals, indicating that the proposed kinematic marker is associated with MRIq in older adults but not in the younger reference group. Importantly, this motor profile was specific: It was not related to cognitive reserve or general cognitive performance, supporting the idea that motor reserve represents a distinct dimension of individual resilience. Within older adults, it was also independent of chronological age, indicating that it captures meaningful inter-individual differences beyond aging itself.

Overall, these findings suggest that motor reserve should not be understood simply as the preservation of speed or strength but as a multidimensional motor profile characterized by stability, lateralization, and consistency across task contexts. The rMR index provides initial evidence for a candidate objective, behavior-based marker of motor reserve in healthy aging. Independent replication in a separate cohort and longitudinal follow-up are required before it can be treated as a validated biomarker.

## Data Availability

The datasets presented in this study can be found in online repositories. The names of the repository/repositories and accession number(s) can be found at: https://data.mendeley.com/datasets/6j7yrhwxjb/1.

## Footnotes

1
http://www.ettsolutions.com

## Glossary

BF₁₀: Bayes factor (evidence for H1 over H0)
BIC: Bayesian information criterion
CONT: continuation task
CPM: colored progressive matrices
CRIq: cognitive reserve index questionnaire
CV: cross-validation
FAB: frontal assessment battery
FDR: false discovery rate
HADS: hospital anxiety and depression scale
HTS: hand test system
IHI: inter-hand interval
IIV: intra-individual variability
IMRL: index–middle–ring–little (tapping sequence)
ITI: inter-tapping interval
LAT: laterality index
LOO: leave-one-out
MaxV: maximum-velocity task
Metro: metronome task
MMSE: mini-mental state examination
MR: motor reserve
MRIq: motor reserve index questionnaire
OLS: ordinary least squares
PCA: principal component analysis
rMR: residual motor reserve index
SPM: self-paced movement task
SUS: system usability scale
TD: touch duration
TE: time error
TMT: trail making test.
