# Supplementary material for: Behavioral markers of motor reserve: stability, lateralization, and consistency
Source: Front Aging Neurosci. 2026 Sep 30;18:1921302. doi: 10.3389/fnagi.2026.1921302 (PMC13661540; doi:10.3389/fnagi.2026.1921302)
Supplement: Supplementary file 1 [file Data_sheet_1.pdf]

ONLINE SUPPLEMENTARY MATERIAL

Behavioral Markers of Motor Reserve: Stability, Lateralization and Consistency

David Vagni[1], Emanuela Principe[2], Melissa Pirrotta[3], Laura Catalano[3], Chiara Milasi[4], Morena De Francesco[4], Andreina Giustiniani[5], Chiara Arena[5], Francesco Scarfone[4], Angelo Quartarone[5], Domenico Bosco[2,4], Giovanni Pioggia[3*], Antonio Cerasa[4*]

S1 Exploratory derivation of the five candidate axes (older subsample, $n = 50$)

The five candidate axes tested in the main analysis were not selected on the full dataset. They were derived from an exploratory analysis of the older subsample alone ($n = 50$), completed in February 2026 and closed before the younger comparison group was recruited between April and June 2026. This section reports that analysis in full, including the strategies that failed. Nothing reported here survived correction for multiple comparisons: the axes rest on convergence across independent analytical strategies, not on any single significant test. MRIq was approximately normally distributed in this subsample ($M = 62.3$, SD = 13.0, range 21.3–98.7) and did not differ by sex ($r_{pb} = 0.012$, $p = .94$).

S1.1 Bivariate correlations

MRIq was correlated with all 597 numeric variables of the dataset, using both Pearson and Spearman coefficients, with Benjamini–Hochberg correction across the full set. Twenty variables reached $p < .05$ uncorrected, with coefficients between |r| = 0.28 and |r| = 0.38; none survived correction, the smallest corrected value being $p_{FDR} = .70$. Eighteen of them were carried forward to the multivariate models of §S1.3. With $n = 50$ and 597 simultaneous tests this is the expected outcome, and the analysis was treated as hypothesis-generating throughout.

The significant variables fell into three functional clusters. The first, and the strongest association in the whole dataset, concerns the variability of touch duration of the dominant hand under self-paced tapping: standard error $r = -0.38$ ($p = .006$) and standard deviation $r = -0.37$ ($p = .008$). Higher lifetime motor activity is associated with more regular self-generated movement durations. The second concerns the non-dominant hand under the metronome: hand rate for all four fingers ($r \approx +0.36$), correct sequences and correct touches ($r = +0.36$), and inter-tap intervals (r between −0.34 and −0.36). The third is sparse and bimanual, and includes the inter-hand offset in the bilateral continuation task ($r = +0.30$).

No variable from the neuropsychological battery reached significance — not the MMSE, nor Rey, Corsi, digit span, Trail Making, Raven, FAB or HADS — and MRIq did not correlate with CRIq ($r = -0.20$). This dissociation, visible already at $n = 50$, is what the discriminant analyses of the main study were designed to test formally.

S1.2 Principal component analysis by task condition

To address the unfavorable ratio between variables and observations, eight separate principal component analyses were run, one per task condition, on z-scored variables, retaining components with eigenvalue > 1 up to a ceiling of ten. This yielded 67 components in total. The structure differs sharply between conditions: the self-paced and maximum-velocity conditions, unimanual and bimanual, are dominated by a single

component explaining between 52% and 60% of the variance, whereas the externally paced conditions are far less compact, with the first component explaining between 22% and 30%. Adding an external pacer decomposes motor performance into more independent dimensions, which is itself part of the rationale for treating paced and unpaced conditions separately.

Six of the 67 components correlated with MRIq at $p < .05$ uncorrected and none survived FDR correction, although the smallest corrected value fell from $p_{FDR} = .70$ in the bivariate analysis to $p_{FDR} = .17$. The six were Bilat_Metro PC10 ($r = +0.43$, $p = .003$), Cont PC10 ($r = −0.35$, $p = .013$), MaxV PC5 ($r = +0.30$, $p = .032$), Metro PC1 ($r = +0.30$, $p = .039$), Metro PC7 ($r = +0.30$, $p = .040$) and SPM PC5 ($r = −0.29$, $p = .044$).

S1.3 Partial least squares regression and multivariate prediction

Partial least squares regression applied directly to each condition, using all glove variables of that condition as predictors (between 53 and 89 variables), failed completely and informatively: cross-validated R^2 was negative in all eight conditions and for every number of components tested, meaning that each model predicted worse than the mean of MRIq. Restricting the predictors to the 18 variables that had reached $p < .05$ in the bivariate analysis produced the first positive cross-validated performance ($R^2_{CV} = 0.10$, $r = +0.33$, $p_{perm} = .002$). Iterative selection by variable importance in projection, starting from all 555 variables and retaining 237, reached $R^2_{CV} = 0.14$, $r = +0.46$.

The strongest model was not the most flexible one. Ordinary least squares regression on the principal components already identified as associated with MRIq gave $R^2_{CV} = 0.17$ and $r = +0.44$ with four components ($p_{perm} = .002$), and $R^2_{CV} = 0.21$ and $r = +0.50$ with six ($p_{perm} = .0025$, $n = 44$). The six standardized coefficients ranged between $|\beta| = 0.17$ and $|\beta| = 0.27$, so no single dimension carried the model. This is the observation that shaped the confirmatory design: aggregating variables into a small number of theoretically motivated indices, rather than letting an algorithm search the variable space, is what recovers predictive signal at this sample size.

S1.4 Derived indices: laterality, between-condition contrasts and intra-individual variability

The dataset does not contain asymmetry, contrast or consistency measures explicitly, so three families of derived indices were computed: 104 laterality indices of the form (right − left)/(right + left) for each measure and condition; 176 contrasts between conditions for the same measure in the same participant, capturing the cost of adding an external constraint; and 79 indices of intra-individual variability across conditions, as standard deviation and coefficient of variation.

The laterality indices produced the strongest associations of the whole derived set, competitive with the best principal components: laterality of touch duration for the middle finger under maximum velocity ($r = −0.42$,

p = .002) and under continuation (r = −0.40, p = .004), and for the four-finger mean under maximum velocity (r = −0.40, p = .004). Eight of 104 reached $p < .05$, with the smallest corrected value of the entire exploratory analysis, p_{FDR} = .13. The between-condition contrasts were weaker (15 of 176 significant, best |r| = 0.35, p_{FDR} = .49). The intra-individual variability indices were intermediate and unusually consistent in direction: 17 of 79 significant, all negative, best |r| = 0.33 for the variability of dominant-hand touch-duration dispersion across conditions (p_{FDR} = .18).

S1.5 Convergence on the five candidate axes

Table S1 and Table S2 summarize the exploratory output. Read across strategies rather than within them, five dimensions recur.

Endogenous timing stability (A1) is supported by the strongest bivariate cluster, by SPM PC5 (β = −0.271 in the six-component model) and by the intra-individual variability of dominant-hand touch duration. Externally paced velocity (A2) is supported by the second bivariate cluster and by Metro PC1 (β = +0.202), the global tapping-speed component. Metro PC7 (β = +0.246) also contributed to the model but loads on inter-tap-interval variability rather than on speed, so it bears on timing rather than on the velocity axis. Bimanual coordination (A3) is supported by the bilateral inter-hand offset and by Bilat_Metro PC10 (β = +0.230), the strongest single component of the analysis. MaxV PC5 (β = +0.171) loads on inter-tap-interval variability in the unimanual maximum-velocity condition and is therefore not itself bimanual evidence. Touch-duration lateralization (A4) is supported by the laterality indices and by Cont PC10 (β = −0.220), which loads on the asymmetry of timing error. Cross-context consistency (A5) is supported by the intra-individual variability family, whose significant associations are uniformly negative (17 of 17), although 11 of the 79 indices in that family correlate positively.

Three criteria then governed the passage from this output to the composites tested in the main study, and they were applied before the younger group was collected. A dimension had to emerge from at least two of the strategies above, so that no axis rests on a single analysis. Its constituent variables had to be directly interpretable raw parameters rather than component scores, which is why no principal component enters any axis despite the components having driven their identification. And the predicted sign had to follow from the way the variables are constructed, so that each axis could subsequently be tested directionally. The resulting composition rules are given in Table 1 of the main text.

Table S1

Exploratory Associations With MRIq in the Older Subsample (n = 50), by Analytical Strategy

Analytical strategy	Tests	$p < .05$	Survive FDR	Best |r|	Best p	Best p_{FDR}
Bivariate correlations (all variables)	597	20	0	0.383	.006	.704
Principal components by condition	67	6	0	0.430	.0025	.169
Laterality indices	104	8	0	0.424	.0022	.132
Between-condition contrasts	176	15	0	0.351	.0124	.485
Intra-individual variability indices	79	17	0	0.333	.0180	.183

Note. FDR correction was applied within each family of tests. No result in any family survived correction, which is why the five candidate axes were treated as hypotheses to be tested on the full sample rather than as findings. The best corrected value improves from p_{FDR} = .70 for single variables to p_{FDR} = .13 for the laterality indices.

Table S2

Multivariate Prediction of MRIq in the Older Subsample (n = 50)

Strategy	Input	Method	R^2 train	R^2_{CV}	r_{CV}	p_{perm}	n
PLS within each condition	53–89 variables	PLS + LOO	0.05–0.67	< 0	—	—	47–50
PLS on bivariate-significant variables	18 variables	PLS + LOO	0.202	0.097	+0.329	.002	50
PLS with iterative VIP selection	555 → 237 variables	PLS + LOO	—	0.143	+0.461	—	43
OLS on four selected components	4 components	OLS + LOO	0.350	0.173	+0.444	.002	44
OLS on six selected components	6 components	OLS + LOO	0.445	0.213	+0.498	.0025	44

Note. R^2_{CV} and r_{CV} are leave-one-out cross-validated. p_{perm} is the permutation *p*-value (2,000 iterations). Sample size varies with listwise deletion on the predictors involved. Negative R^2_{CV} means that the model predicts worse than the mean of MRIq.

Methods

2.4 Data preprocessing

Preprocessing followed the steps below:

2.4.1 Variable cleanup

Two variables were removed prior to analysis: one empty column carried over from the export pipeline and the raw Ideational Apraxia score, which had zero variance across the sample. The raw

Ideomotor Apraxia score was retained despite near-zero variance (M = 19.96, SD = 0.28, and no variance at all within the older group); it enters no analysis reported here.

2.4.2 Reconstruction of the Continuation task counters

Inspection of the count-based variables of the Continuation task (TOTAL_TOUCHES, CORRECT_SEQUENCES, total correct touches, and IHI counters) revealed an internal inconsistency in the younger-adult subsample: the ratio total_touches / (hand_rate × 60 s) was approximately 0.49 (*SD* 0.013) in younger participants versus approximately 0.99 (*SD* 0.07) in older participants and approximately 0.98 in the same subjects' other tasks (SPM, MaxV, Metro). The exact halving and the disappearance of biological variability indicated that, in the younger batch, count-based variables had been exported on the post-metronome 30-s continuation phase only, whereas in the older batch they reflected the full 60-s task. We retrieved the raw glove output (CSV, two rows per Continuation task in younger participants: one for the synchronization phase, one for the continuation phase) and aggregated the two phases to reconstruct the 60-s window, applying: sum for count variables; weighted means (with sequence counts as weights) for finger-level means and rates and timing-error means; pooled-variance formulas for standard deviations and standard errors; and recomputation of TD/ITI ratios from the aggregated TD and ITI. After this correction, the consistency check returned ratios of approximately 0.98 in both groups across all four hand-task combinations, matching the other tasks.

2.4.3 Selective masking of invalid data

Four cases of clearly invalid data were addressed by masking the affected entries (rather than excluding the participants altogether):

- Subject ID35 had impossible right-hand metronome data: mean ITI of −60 ms with all individual finger ITIs equal to zero, and full missingness on time-error variables. The right-hand metronome variables were masked for this subject; the corresponding left-hand and bilateral conditions were retained.
- Subjects ID6 and ID44 had identical anomalous total-time-elapsed values for the left-hand continuation task (67,763 ms vs. ~60,000 ms in all other subjects/conditions); the total-time-elapsed variable for that condition was masked for these two subjects.
- Subject ID23 had a non-plausible Edinburgh score of 1.05 (the inventory yields integers or half-integers); the score was rounded to 1.0.
- One older subject had a raw SUS of 44, exceeding the 0–40 raw scale; the converted score (110, beyond the 0–100 range) was capped at 100.

2.4.4 Removal of mathematically derived variables

An audit of the 555 motor variables identified 133 redundant or near-redundant variables to be removed, in order to avoid spurious multicollinearity in subsequent multivariate models. Of these, 122 were exact mathematical derivations of other variables (pairwise correlations $|r| \geq 0.999$, coefficient of variation of the ratio < 1%): (i) total correct touches equal to four times the count of correct sequences ($n = 23$); (ii) per-finger hand rates that replicated the hand-level rate, the IMRL cycle frequency being identical for the four fingers by design ($n = 38$); (iii) the ratio TD/ITI computed on a per-cell basis ($n = 53$); (iv) bilateral IHI counters duplicated across hands ($n = 4$); (v) two columns whose nominal label and content were inconsistent (originally labeled "DX" but containing values of "SX"; $n = 2$); (vi) two further columns whose nominal label suggested a TDITI ratio but whose values matched the corresponding hand rate ($n = 2$). A further 11 variables were standard errors very tightly correlated with the corresponding standard deviations ($r \approx 0.97$); these are not exact derivations but were treated as near-redundant and removed for the same reason. The mean-of-fingers indices (TD_MEAN, ITI_MEAN; $n = 15$) were preserved because they served as the basis for the laterality indices in §2.6. The cleaned dataset comprised 422 informative motor variables.

3. Results

3.7 Sensitivity and robustness analyses

The direction and significance of the rMR × MRIq association within the older group were stable across alternative specifications (Table S3). The Pearson magnitude, by contrast, was sensitive to one high-leverage observation, treated separately below. Restricting the analysis to strict right-handers (excluding 3 mixed-handed subjects) yielded $r = +0.57$; excluding three multivariate outliers (subjects with > 40 motor variables at $|z| > 3$: ID35, ID40, ID19) yielded $r = +0.57$; excluding only subject ID35 (with masked right-hand metronome data, see §2.4.3) yielded $r = +0.57$. In a multiple regression with age, sex, and education as covariates, the rMR coefficient remained highly significant ($\beta_{unstd} = +9.09$, $p < .001$) and none of the covariates reached significance (gender $p = .73$, education $p = .54$, age $p = .13$). The Huber robust regression coefficient (βstd = +0.543) was almost identical to the OLS coefficient (βstd = +0.565) and to the bounded-influence MM-estimate (βstd = +0.540). Replacing Pearson with Spearman, Kendall, rank-transformed, or log-transformed measures preserved the significance of the association in every case (range of r/ρ/τ from +0.32 to +0.60, all $p \leq .002$).

High-leverage point. OLS influence diagnostics flagged subject ID29 as a high-leverage observation in the bivariate rMR × MRIq plane within the older group: leverage $h_{ii} = 0.32$ (≈ 4× the conventional 2p/n threshold), Cook's $d = 0.48$ (≈ 6× the 4/n threshold), DFFITS = −0.99 (≈ 2.5× the $2\sqrt{(p/n)}$ threshold). The studentized residual was −1.41, so ID29 lies on the regression line rather than in the y-tail: the case is the

most extreme combination of low MRIq ($z = -3.15$ within the older group) with high A1 timing endogenous ($z = +3.56$) and high A5 cross-context IIV ($z = +3.17$), with no anomaly in age, education, neuropsychological profile, mood (HADS-A = 4, HADS-D = 1), or task usability (SUS = 70). We retained ID29 in the main analysis because the case represents the extreme tail of a real distribution rather than a measurement artifact. The impact of its exclusion is reported next. Removing ID29 reduces the Pearson magnitude to $r = +0.42$ (parametric $p = .003$, permutation $p = .0014$; bootstrap 95% CI [+0.20, +0.59]) and the cross-validated R^2 to $R^2_{LOO} = 0.11$ ($r_{LOO} = +0.34$). The Spearman correlation, which is invariant to high-leverage points in x, is virtually unchanged (ρ = +0.46 → +0.43). A second observation (ID26) also exceeded the conventional Cook's *d* threshold (Cook's $d = 0.16$, against $4/n = 0.080$), this time on the opposite side of the regression: high MRIq ($z = +2.79$ within the older group) paired with moderately positive rMR ($z = +1.09$). Excluding both ID29 and ID26 yielded $r = +0.39$ (Table S3), confirming that the bivariate association is not carried by either single high-leverage point. The MM-estimator with Tukey biweight (a bounded-influence robust estimator that targets high-leverage points, unlike the Huber M-regression which targets large residuals) yielded $\beta_{std} = +0.54$, close to OLS. The OLS Pearson estimate on $n = 50$ should therefore be read as an upper bound on effect size, with the Spearman $\rho \approx +0.43$–0.46 and the MM-estimator $\beta_{std} = +0.54$ as more conservative central estimates.

Table S3

Sensitivity of the rMR × MRIq Association Within the Older Subsample Under Alternative Specifications

**Sensitivity specification**	***N***	***r* Pearson**	***p***	**ρ Spearman**	**R^2_{LOO}**
Reference (within the older group)	50	+0.565	< .001	+0.461	0.25
Strict right-handers only	47	+0.568	< .001	+0.45	0.25
Excl. multivariate outliers (ID35, ID40, ID19)	47	+0.570	< .001	+0.49	0.25
Excl. ID35 only	49	+0.572	< .001	+0.49	0.25
Excl. ID29 (high-leverage, Cook's *d* > 4/n)	49	+0.421	.003 / .0014^{+}	+0.427	0.11
Excl. ID29 + ID26 (both Cook's *d* > 4/n)	48	+0.389	.006	+0.396	0.08
MM-regression (Tukey biweight)	50	$\beta_{std} = +0.54$	< .001	—	—
Adjusted for age + sex + education	50	$\beta_{unstd} = +9.09$	< .001	—	—

Note. ID29 is a high-leverage point retained in the main analysis; excluding it reduces the Pearson magnitude but not the direction or significance of the association, and leaves the rank-based Spearman essentially unchanged. The MM-estimator with Tukey biweight is reported because the standard Huber M-regression

downweights large residuals but does not target high-leverage points. Where two *p*-values are reported (e.g., .003 / .0014+), the first is the parametric Pearson *p* and the value followed by + is the permutation *p* (1,000 sign-flip permutations on residuals).

Table S4

Sensitivity of the Residual Motor Reserve Index to the Form of the Age Baseline

Baseline model for the age trajectory	*r* (older group)	R^2(LOO)	*r* with linear version
Linear (pre-specified)	+0.567	0.249	1.000
Theil–Sen (robust)	+0.562	0.241	0.999
Huber M-estimator	+0.565	0.245	1.000
Quadratic age term	+0.582	0.266	0.995
Intercept-only (age term removed)	+0.563	0.243	1.000
Pooled across both groups	+0.571	0.253	1.000

Note. For each specification the younger-group model was re-estimated, residuals were recomputed for the three retained axes (A1, A4, A5), sign-oriented and averaged, and the resulting index correlated with MRIq within the older group. The last column reports the correlation between each version of the index and the pre-specified linear version. The intercept-only row removes the age term altogether: the association is essentially unchanged, which shows that in this bimodal design the residualization operates close to a standardization against the younger mean.

Threshold analysis. Segmented regression fitted the data better than a single-line model ($F(2,46) = 6.26$, $p = .004$), with the optimal knot at rMR = 0.85. Pairwise comparisons across MRIq tertiles (Mann–Whitney) showed that the low-MRIq tertile differed from both the mid-MRIq ($U = 70$, $p = .018$) and high-MRIq ($U = 71$, $p = .012$) tertiles. Mid- and high-MRIq tertiles did not differ ($U = 130$, $p = .84$). The Spearman ordinal trend across tertiles was significant ($\rho = +0.37$, $p = .009$) and the Kruskal–Wallis omnibus test confirmed group differences ($H = 8.20$, $p = .017$). The pattern points to a threshold effect: the residual reserve index discriminates low from non-low motor reserve, but not fine gradations within the high range. The segmented model rests on only five points above the knot and should therefore be treated as exploratory until replicated in a larger older-adult sample.

3.8 Anatomical specificity of the lateralization effect

Jackknife analysis on the three robust axes within the older subsample identified A4 (TD lateralization) as the dominant driver: against a baseline of $R^2_{LOO} = 0.20$ for the three-axis model, removing A1 left performance unchanged (0.20) and removing A5 barely altered it (0.19), whereas removing A4 reduced it to 0.06. Taken alone, A4 reaches $R^2_{LOO} = 0.14$, against 0.05 for A5 and 0.03 for A1. To dissect A4 further,

we computed the laterality index of touch duration separately for each finger (index, middle, ring, little, plus the four-finger mean) and each task condition (SPM, MaxV, Metro, CONT), and tested its association with MRIq within the older subsample; the full condition × finger matrix is reported as Figure 7 of the main text.

The strongest correlations appeared for the middle finger in the maximum-velocity task ($r = -0.42$, $p = .002$) and in the continuation task ($r = -0.40$, $p = .004$), and for the four-finger mean in the same two tasks ($r = -0.40$ in MaxV, $r = -0.35$ in CONT). The ring finger contributed at intermediate strength in both MaxV and CONT ($r \approx -0.35$); the little finger contributed only in CONT ($r = -0.34$); the index finger contributed only weakly. The self-paced and metronome conditions did not produce significant lateralization effects for any finger. Seven of the twenty finger × task correlations survived Benjamini–Hochberg FDR correction across the family, all in the maximum-velocity and continuation tasks. The effect is concentrated on the middle finger and its neighbors under demanding pacing (maximum velocity and continuation), which fits the larger cortical representation of the middle finger and the larger effect of motor demand on dominant-vs-nondominant asymmetry.

Discussion

Trait stability and threshold structure

Two further features of the residual reserve effect deserve discussion. First, rMR did not decline with chronological age within the older group ($r = -0.09$ with age across 50–76 years), and the same held for each component axis. The interpretation requires care. By construction, rMR removes the linear age component derived from the younger baseline, so the within-older-group age coefficient should be small whenever that linear projection captures most of the systematic age-related variance in the older range. Independent evidence indicates that within-older fine motor performance does correlate cross-sectionally and longitudinally with motor-tract microstructure, particularly the forceps minor (Oschwald et al., 2021): some age-graded variance in motor performance survives within the older adult range, and a strictly age-flat residual is therefore a property of our residualization choice rather than a substantive proof of trait stability. What we can say more cautiously is that the inter-individual variation captured by rMR within 50–76 years is not dominated by further linear age regression; it characterizes *which* individuals diverge from the younger-baseline projection at their age, rather than tracking how much they have lost as a function of how old they are.

Second, the relationship between rMR and MRIq fits a threshold pattern. Non-parametric comparisons across MRIq tertiles showed a significant difference between the low tertile and both the mid and high tertiles, with no difference between mid and high. Segmented regression fitted the data better than a single-

line linear model ($F(2,46) = 6.26$, $p = .004$), with a knot at rMR ≈ 0.85. The residual reserve index discriminates low motor reserve from the rest, while finer gradations within the high-reserve range are less well separated. The pattern resembles a step function in which the protective effect of motor reserve plateaus above a sufficient level. The segmented model is sensitive to the small number of points above the knot ($n = 5$), and the effect should be read as exploratory until replicated.

Motor reserve as a distinct construct

The lack of correlation between rMR and either CRIq or the neuropsychological composite, confirmed by Steiger's z-tests ($z > 3.3$, $p < .001$ in both contrasts), supports a construct-level distinction between motor reserve and cognitive reserve. The pattern is coherent with the modest, slightly negative within-group correlation we observed between MRIq and CRIq (Old: $r = -0.20$; Young: $r = +0.19$). These correlations are weak, non-significant, of opposite sign across groups, and, given the restricted younger CRIq range, do not support a life-history trade-off; we note only that high cognitive reserve and high motor reserve need not coincide in the same individual. The residual reserve index captures a dimension that is neither a generic "healthy aging" axis nor a cognitive-performance axis, but a motor-performance reserve axis. A converging finding comes from Castelli et al. (2020), who showed in patients with multiple sclerosis that premorbid physical activity and cognitive reserve modulate rehabilitation outcome in a strictly intra-modal way: physical-activity reserve predicts response to balance training, cognitive reserve predicts response to attention training, with no cross-domain transfer. The dissociation between motor and cognitive reserve we report in healthy aging fits the same domain-specific architecture. The discriminant pattern matches theoretical accounts in which reserve is multidimensional and is supported by partly distinct neural substrates (Cabeza et al., 2018; Stern et al., 2020); recent work in Parkinson's disease has extended the same framework to the motor domain explicitly (Hoenig, 2026). Motor reserve may share with cognitive reserve a common prefrontal-cognitive-control component, but the orthogonality we observe between rMR and both CRIq and the neuropsychological composite implies a substantial contribution from motor-specific circuits, plausibly cortico-cerebellar and basal-ganglia networks involved in timing and motor pacing, that future neuroimaging work should test directly.

Distinction from the motoric cognitive risk syndrome. Our finding of orthogonality between rMR and the neuropsychological composite must be reconciled with the literature on the motoric cognitive risk syndrome (MCR; Verghese et al., 2013), in which slow gait paired with subjective cognitive complaints predicts incident dementia. The two constructs are concentrating on different signals. The MCR captures absolute motor slowing in older adults, the same age-shared decline that our Simpson check explicitly removes. The rMR index, by construction, is the variability in motor function that *cannot* be attributed to chronological aging. The expected behavior of the two indices is therefore complementary rather than

redundant: MCR rises with cognitive deterioration because both gait and cognition decline in concert with brain aging, while rMR is informative precisely about the inter-individual differences that survive the aging-shared variance. The null association between rMR and the neuropsychological composite is consistent with this conceptual separation.

As a measurement instrument, an objective behavioral index of motor reserve obtained from a 12-minute glove-tapping protocol converges with the self-report MRIq within older adults while remaining discriminant against cognitive reserve and cognitive performance. To our knowledge, MRIq has not previously been triangulated against an objective motor measure of this kind; the residual approach fits the task because it builds the requirement of informativeness beyond aging directly into the index.

Methodological Implications and Future Perspectives: Methodologically, the residual reserve approach inherited from cognitive reserve research transfers to motor measures *provided that* candidate biomarkers are pre-aggregated into theoretically motivated composites before residualization. Our supplementary analyses confirm a known property of the $p \gg n$ regime: data-driven model selection on the full set of motor variables (elastic net, ridge, random forest) produced cross-validated R^2 close to or below zero in the older subsample, regardless of regularization, while the pre-specified composites reached $R^2_{CV} \approx 0.20–0.25$. Theory-guided dimensionality reduction is not a stylistic preference here but a practical necessity for biomarker discovery.

Three directions are worth pursuing. The rMR index should be validated in an independent cohort, ideally with continuous age sampling across the adult lifespan, to characterize its trajectory and assess whether the age-independence we observe within the older range corresponds to genuine longitudinal stability. Sub-scale analyses of MRIq, testing whether sport, occupational, or leisure-related items differentially track rMR, would clarify *which* life-history experiences contribute most to the objective motor profile. Application to clinical populations is the natural translational extension: the motor reserve framework, like the cognitive reserve framework before it, gains its translational value when it explains *differential trajectories of decline* in conditions where motor function is at risk: Parkinson's disease, mild cognitive impairment with motor symptoms, multiple sclerosis, and post-stroke recovery. The framework has accumulated converging evidence in Parkinson's disease, where premorbid physical activity and educational attainment have each been associated with reduced motor burden at matched levels of dopaminergic depletion (Sunwoo et al., 2016; Blume et al., 2017), and where baseline motor reserve estimated on dopamine-transporter imaging in 303 de novo PD patients followed for nearly nine years predicts slower progression of motor, cognitive and non-motor symptoms (Bai et al., 2024) and the rate of progression itself (Lee et al., 2025). The case for an application to mild cognitive impairment (MCI) is reinforced by longitudinal evidence that gait speed and finger-tapping speed begin to decelerate up to 12 years before the clinical onset of MCI (Buracchio et al., 2010), making early motor markers a plausible window onto pre-

clinical decline. The natural testbed for rMR is therefore the pre-clinical or compensated phase of these conditions, where a behaviorally-derived reserve index can be tested against differential trajectories of decline.
